# Evidence that variation in root anatomy contributes to local adaptation in Mexican native maize

**DOI:** 10.1111/eva.13673

**Published:** 2024-03-10

**Authors:** Chloee M. McLaughlin, Meng Li, Melanie Perryman, Adrien Heymans, Hannah Schneider, Jesse R. Lasky, Ruairidh J. H. Sawers

**Affiliations:** ^1^ Intercollege Graduate Degree Program in Plant Biology The Pennsylvania State University University Park Pennsylvania USA; ^2^ Department of Plant Science The Pennsylvania State University University Park Pennsylvania USA; ^3^ Umeå Plant Science Centre, Department of Forest Genetics and Plant Physiology Swedish University of Agricultural Sciences Umeå Sweden; ^4^ Earth and Life Institute UC Louvain Louvain‐la‐Neuve Belgium; ^5^ Department of Physiology and Cell Biology Leibniz Institute for Plant Genetics and Crop Plant Research (IPK) Seeland Germany; ^6^ Department of Biology The Pennsylvania State University University Park Pennsylvania USA

**Keywords:** GEA, local adaptation, maize, root anatomy

## Abstract

Mexican native maize (*Zea mays* ssp. *mays*) is adapted to a wide range of climatic and edaphic conditions. Here, we focus specifically on the potential role of root anatomical variation in this adaptation. Given the investment required to characterize root anatomy, we present a machine‐learning approach using environmental descriptors to project trait variation from a relatively small training panel onto a larger panel of genotyped and georeferenced Mexican maize accessions. The resulting models defined potential biologically relevant clines across a complex environment that we used subsequently for genotype–environment association. We found evidence of systematic variation in maize root anatomy across Mexico, notably a prevalence of trait combinations favoring a reduction in axial hydraulic conductance in varieties sourced from cooler, drier highland areas. We discuss our results in the context of previously described water‐banking strategies and present candidate genes that are associated with both root anatomical and environmental variation. Our strategy is a refinement of standard environmental genome‐wide association analysis that is applicable whenever a training set of georeferenced phenotypic data is available.

## 1 INTRODUCTION

Abiotic stress is a major driver of plant phenotypic diversity (Lowry, 2012), acting to select locally adapted varieties with specific morphological, physiological, and phenological traits (Fumagalli et al., 2011; Hereford, 2009; Stebbins, 1952). Differences in such selective pressures over a continuously varying environment produce clines of genetic and phenotypic variation, reflecting the shifting costs and benefits of diverse biological strategies (Joswig et al., 2022). Although plants are plastic in the face of environmental challenges (Des Marais et al., 2013; Lasky et al., 2012, 2014), locally adapted specialists can constitutively express adaptive strategies, anticipating the need for the induction of stress responses (Aguilar‐Rangel et al., 2017; Levins, 1968; von Heckel et al., 2016). As a consequence, genotypes sourced from diverse locations can still display trait variation indicative of adaptation to their home environments when grown in a benign common garden (Janzen et al., 2022; Shimono et al., 2009; Stinchcombe et al., 2004).

Mexican native maize (*Zea mays* ssp. *mays*) varieties (“landraces”) represent an attractive system for the study of local adaptation. Mexico is the center of origin of maize, and today hosts 59 described native varieties cultivated from sea level to an elevation of 3400 m, in environments ranging from semi‐arid to hot and humid (Arteaga et al., 2016; Perales & Golicher, 2014; Ruiz Corral et al., 2008). This diversity has been extensively sampled, and large collections of georeferenced and genetically characterized material are available (Arteaga et al., 2016; Janzen et al., 2022; Mercer & Perales, 2019; Romero Navarro et al., 2017). Throughout maize domestication and diversification, farmers have consciously selected for agronomically and culturally desirable traits, notably targeting the female inflorescence (the ear), to produce a rich variety of forms (Bellon et al., 2018; Louette & Smale, 2000). In parallel, a likely unconscious process of selection has acted to adapt varieties to local conditions (Janzen et al., 2022; Mercer & Perales, 2019; Romero Navarro et al., 2017) and enhance tolerance to different environmental stressors after dispersal to new environments (Eagles & Lothrop, 1994; Magalhaes et al., 2007).

In this study, we focus specifically on the potential role of root trait variation in the adaptation of maize to different climatic and edaphic environments in Mexico. Although less easily visible than aboveground traits, the maize root system has been substantially impacted by domestication, diversification, and modern breeding (Burton et al., 2013; Chen et al., 2022; Gaudin et al., 2014; Lopez‐Valdivia et al., 2022; Ren et al., 2022). Roots are fundamental to plant water and nutrient acquisition and play a key role in both wild and domesticated plants in determining performance under resource limitation (Ma et al., 2018; Markesteijn & Poorter, 2009; Wahl & Ryser, 2000). Within maize specifically, root trait variation among inbred breeding lines has been linked to performance differences under both water (Bomfim et al., 2011; Jaramillo et al., 2013; Schneider et al., 2020) and nutrient (Galindo‐Castañeda et al., 2018; Schneider, Postma, et al., 2017) limitation. Extensive root trait variation has also been reported among native maize varieties (Bayuelo‐Jiménez et al., 2011; Berauer et al., 2023; Burton et al., 2013), although the associated functional impact and possible adaptive roles remain to be fully characterized. Variations in the plant root system can be considered from the anatomy of individual roots to overall root system architecture (Jung & McCouch, 2013; Lynch, 2019), with traits at all levels interacting to determine overall root system function in the context of a given environment (Klein et al., 2020). Here, we limit ourselves to consideration of variation in root anatomy.

The maize root system consists of a variety of root classes that vary in function and importance during development (Atkinson et al., 2014; Hochholdinger, 2009; Viana et al., 2022). Within this range, root anatomy develops on a basic pattern of radially organized tissue types: an external epidermis, the ground tissue, and an inner stele containing the pericycle and vasculature (Lynch et al., 2021). The epidermis protects the inner layers from physical damage and is in direct contact with the rhizosphere, playing a key role in water and nutrient exchange. The ground tissue is further differentiated into the cortex and the endodermis that encloses the stele. In the model plant *Arabidopsis*, the cortex is composed of only two layers, a single layer of cortical parenchyma and the endodermis. In maize, however, the cortex divides to form multiple cell layers, impacting the physical (Chimungu et al., 2015), hydraulic (Heymans et al., 2020; Sidhu et al., 2023) and radial nutrient transport (Hu et al., 2014; Schneider, Wojciechowski, et al., 2017) properties of the root, as well as accommodating beneficial endomycorrhizal fungi (Bennett & Groten, 2022; Sawers et al., 2008). Developmental and environmental cues can trigger cells in the cortex to undergo programmed cell death and form aerenchyma. The resulting cortical air‐filled lacunae help maintain gas exchange and mitigate hypoxia under flooding (Colmer, 2003; Mano & Nakazono, 2021). In addition, the reduction in root metabolic cost following aerenchyma formation can be beneficial in resource‐limiting conditions including drought and low availability of nitrogen or phosphorus (Galindo‐Castañeda et al., 2018; Jaramillo et al., 2013). The cell walls of the endodermis are impregnated with suberin to form the Casparian strip and, in later development, further reinforced with lignin to act as a barrier that restricts apoplastic transport into the stele from the surrounding cortical root tissue. The central stele contains the xylem and phloem vessels that axially transport water and nutrients. The xylem is composed of small protoxylem vessels and larger metaxylem vessels, the latter providing most of the transport capacity in mature root tissues (Doussan et al., 1998). The size and number of metaxylem vessels and living cortical area influence root radial and axial hydraulic properties (conductivity and conductance; Frensch & Steudle, 1989; Schneider, Wojciechowski, et al., 2017), impacting water capture and plant performance (Couvreur et al., 2012; Richards & Passioura, 1989).

In this study, we aim to characterize heritable variation in root anatomical traits in Mexican native maize and to associate patterns of phenotypic and genetic variation with the source environment. The genetic basis of local adaptation can be characterized through associations between genotype and phenotypes involved in local adaptation or between genotype and environment (Fournier‐Level et al., 2011; Hoban et al., 2016). Approaches using genotype‐environment association (GEA) have the advantage of not requiring resource‐costly phenotypic characterization, although they must assume local adaptation to have occurred over the tested environments and can be complicated by the confounding effects of population structure (Lasky et al., 2023). One challenge of GEA is that it can be unclear which aspect(s) of the environment might drive selection, such that it may be difficult to know which associations to test. Furthermore, although GEA can identify variants changing in frequency over the environment, the approach provides no direct insight into the nature of any associated traits and adaptations.

To study local adaptation in the root anatomy of Mexican native maize, we sought a methodology to focus the standard GEA approach toward predefined traits of interest. Although, as described above, extensive genotype‐environment data is available, only a small fraction of total maize diversity has been directly evaluated for root anatomy and, even with investment beyond the scope of this study, this situation is unlikely to change. In consequence, we present an approach that first fits trait‐environment relationships using a relatively small training set of phenotypically characterized, georeferenced varieties. These fitted models are then used to project trait values onto a larger set of varieties for which genotypic and georeference – but not root anatomical – data are available, and these predicted trait values are used in turn for genome‐wide association (GWA). We compare the results of our approach with standard GEA and a previously published root anatomy GWA based on phenotypic characterization of modern maize breeding lines. Our approach provides evidence for systematic variation in root anatomy tracking environmental differences across the Mexican landscape, identifying candidate genes associated with both phenotypic and genetic clines.

## 2 MATERIALS AND METHODS

### 2.1 Data processing and statistical analysis

R statistical software (R Core Team, 2022) was used throughout for data processing and statistical analysis as detailed below.

### 2.2 Phenotyped Burton panel

Phenotypic data from a previous characterization of greenhouse‐grown native Mexican maize were obtained from Burton et al. (2013) (Supporting Files in Data S1). This study describes root anatomical variation in axial roots from the second node of traditional maize varieties and teosintes. After filtering for traditional maize accessions of Mexican origin, we used 39 georeferenced samples for our subsequent analyses. The traits *total metaxylem vessel area* (MVA), *individual metaxylem diameter* (MD), *individual metaxylem area* (MA), *number of metaxylem vessels* (NMV), and *cortical cell size* (CCS) were not included in the original report and were obtained here by re‐analysis of the cross‐section images using *RootScan* v2.4 imaging software (Burton et al., 2012).

### 2.3 Genotyped CIMMyT panel

Genotypes from a collection of 1791 native Mexican maize accessions from the CIMMyT Maize Germplasm Bank (CIMMyT panel) were obtained from Romero Navarro et al. (2017) and Gates et al. (2019). In brief, sequences were generated using Illumina HiSeq, and genotypes were called in TASSEL. Missing SNPs were imputed using BEAGLE4, and SNPs were further filtered for minor allele frequency >1%. The genotype data was uplifted to coordinates on the B73 v4 reference genome using Crossmap.

### 2.4 Environmental data

We compiled climatic and soil data for the source location of both the Burton and CIMMyT panel varieties from publicly available databases (Supporting Files in Data S1). Climate data was extracted using R/raster::extract (Hijmans, 2023) following the methods described in Lasky et al., 2015. Monthly minimum, maximum, and mean temperatures, mean monthly precipitation, and other derived parameters of biological importance that consider temperature and precipitation dynamics were taken from WorldClim (Hijmans et al., 2005). Monthly and annual average potential evapotranspiration (PET), and a measure of aridity (mean annual precipitation divided by mean annual PET) were from the CGIAR‐CSI Globality‐Aridity database (Zomer et al., 2008). Information on inter‐annual variability in precipitation was calculated with data from the NCEP/NCAR Reanalysis project (https://psl.noaa.gov/data/reanalysis/reanalysis.shtml; Kalnay et al., 1996). Inter‐annual variability in precipitation was obtained by calculating each calendar month's coefficient of variation (CV) across years for each month's surface precipitation rate. Information on estimated photosynthetically active radiation (PAR) for each quarter was averaged for data collected from NASA SRB (https://asdc.larc.nasa.gov/project/SRB). Vapor pressure deficit (VPD) was taken from the Climate Research Unit (New et al., 2002). Soil data was from SoilGrids (Hengl et al., 2017) and the Global Soil Dataset (GSD; Shangguan et al., 2014). Data from GSD includes soil features of the topsoil and 1 m below the surface. We found a strong correlation between values for topsoil and 1 m below the surface (for all variables, correlation of >70%) and excluded the topsoil data from our dataset. All soil variables were cleaned by removing outliers missing values imputed using the MICE package (Fox et al., 2017; van Buuren & Groothuis‐Oudshoorn, 2011).

### 2.5 GRANAR representation and MECHA estimation of emergent hydraulic properties

Generator of Root Anatomy in R (GRANAR; https://granar.github.io) and the Model of Explicit Cross‐section Hydraulic Architecture (MECHA; https://mecharoot.github.io) are open‐sourced computational tools. GRANAR uses anatomical parameters as inputs to generate digital root anatomies. Once constructed, GRANAR root anatomies can be used for digital visualizations of anatomical parameters, and the anatomical network can be written as an XML file with the same format as CellSet output (Pound et al., 2012). MECHA uses GRANAR anatomies to estimate hydraulic properties. We used GRANAR to reconstruct virtual anatomies for all observed accessions of the Burton panel, our predictions of the Burton panel Mexican lines, predicted CIMMyT clusters, and predicted and observed novel germplasm is grown in this study. As the *root cross‐section area* was not predicted by our random forest (RF) models, we used the Burton panel mean for our predicted GRANAR models. Similarly, the *total stele area* was not directly predicted but calculated from the predicted *total stele area*: *root cross‐section area* using the Burton panel *root cross‐section area* mean. We estimated the root hydraulic conductance (*K*_r_ and *K*_x_) and conductivity (*k*_r_) with MECHA. The subcellular hydraulic parameters are the same as in Heymans et al. 2020, and the chosen hydraulic scenario accounts for the hydrophobic structures of an endodermal Casparian strip. The script used is available on a GitHub repository HydraulicViper/RootDiversity (doi: 10.5281/zenodo.10104521) under a GPL‐3 license.

### 2.6 Principal component analysis

Principal component analysis (PCA) was conducted with R/ade4::dudi.pca (Dray & Dufour, 2007) and visualized with R/factoextra (Kassambara & Mundt, 2020). Correlation of the first three phenotypic PC loadings and calculated hydraulic properties to features of source environment (elevation, annual precipitation, annual mean temperature, and soil pH) was evaluated in R, and *p*‐values were adjusted for multiple testing using the Holm method with R/stats::p.adjust (*α* = 0.05).

### 2.7 Random forest model prediction

RF is a non‐parametric classification method that constructs decision trees using subsets of input data to select the predictor variables that limit variance for the response variable predictive model (Breiman, 2001). RF models have been found to have high predictive performance when tested data include a large number of predictor variables with, individually, little relationship to response variables (Fox et al., 2017); however, best practice is to limit the number of predictor variables to avoid model overfitting. To select informative features for Random Forest (RF) modeling, we filtered the set of environmental descriptors using the function R/Boruta::boruta (Kursa & Rudnicki, 2010) to retain those showing some relationship with each of our root anatomical traits. As the environmental variables used in this study were continuous, RF models were built as regression trees. RF models were built using R/randomForest::randomForest (Liaw & Wiener, 2002), 5000 trees were built per model and one‐third of the number of explanatory variables were tried at each split. We increased our number of trees from the default value (500) to account for models with a large number of predictors. Model success was evaluated as the percent variance explained and the correlation coefficient between observed and RF‐predicted trait values. The contribution of each environmental descriptor in a given RF model was quantified using SHapley Additive exPlanations (SHAP) values (Lundberg & Lee, 2017). RF models were used to predict variation in nine anatomical traits for the 1791 georeferenced and genotyped accessions in the CIMMyT panel with R/caret::predict (Kuhn, 2021).

### 2.8 Clustering analysis

CIMMyT panel accessions were clustered by predicted root anatomical traits using Partitioning Around Medoids (PAM) as previously described (Klein et al., 2020). Briefly, anatomical trait values were centered and scaled using R/caret (Kuhn, 2021), and outlying values (>3 standard deviations from the mean) were removed. Within cluster sums of squares (WSS) were visualized as a function of cluster number using R/factoextra::fviz_nbclust (Kassambara & Mundt, 2020). From inspection of the resulting curve, the accessions were grouped into seven clusters using R/cluster::pam (Maechler et al., 2021) under default settings. Primary variety (landrace) designations were assigned using data available from CIMMyT (www.mgb.cimmyt.org; 1454 accessions assigned), and these matched to existing morphological‐isozymatic (Sanchez et al., 2000) and environmental (Ruiz Corral et al., 2008) classifications. Testing for enrichment of a given variety in a given cluster was performed using Fisher tests with R/stats::fisher.test, under a contingency table formed by partitioning the 1454 accessions by membership of the cluster and assignment to the variety. Results were adjusted using the Holm method with R/stats::p.adjust (*α* = 0.05).

### 2.9 Greenhouse evaluation of root anatomy

We selected eight native Mexican maize accessions, representative of environmental diversity within the CIMMyT panel and that had not been previously evaluated for root anatomical traits, for phenotypic validation of RF anatomical predictions. Ten biological replicates of each accession were grown in a greenhouse in State College, PA (40.8028708, −77.8640406) from April to May of 2022. Plants were grown in 2.83 L pots (4 in × 4 in × 14 in, Greenhouse Megastore). The growth media was a mix of silica sand (50%), turface (30%), and field soil (20%) sourced from Rock Springs, PA. Pots were watered to field capacity the night before planting and watered again to field capacity every day after sowing until germination. Once germinated, plants were watered with 200 mL of tap water every other day. One week after germination, plants were fertigated with Peters Excel 15 – 5 – 15 Cal Mag Special with Black Iron 200 ppm N recipe and supplemented with an extra 5 ppm Fe (Sprint 330), fed at 1:100 dilution, two times per week until harvest. Greenhouse settings were set at 16‐h days, with a minimum temperature of 21°C and a maximum temperature of 28°C.

Following Burton et al. (2013), 28 days after planting, plants were destructively harvested. Two representative axial roots from the second and third root nodes were collected. From each axial root, a 4‐cm root sample was excised 5–9 cm from the most basal portion of the sample. Root samples were stored in 75% ethanol until sectioned by laser ablation tomography (LAT; Strock et al., 2022). In LAT, a sample is moved via an automatic stage towards a 355‐nm Avia 7000 pulsed laser and ablated in the focal plane of a camera. A Canon T3i camera with a 53micro lens (MP‐E 65 mm) was used to capture images of the root cross‐section. Two representative images for each root sample, sectioned 1–3 cm apart, were saved for later image analysis with *RootScan* v2.4 imaging software (Burton et al., 2012). Anatomical phenotypes were averaged for each nodal root of a plant, where each value is an average of two roots from each node and two LAT image sections of each root. We used linear mixed models with R/lme4 (Bates et al., 2007) to calculate the best linear unbiased predictions (BLUPs) for each trait in each accession as:

$${\text{Trait}}_{\mathrm{rwt}}=\beta_{0}+R_{\text{0r}}+N_{\text{0n}}+T_{\text{0t}}+{\text{34𝑒}}_{\mathrm{rnt}}$$

where for each modeled trait, Trait_rwt_, *β*_0_ is the overall intercept, *R*_0r_ is the random effect of accession, *N*_0n_ is the random effect of node, *T*_0t_ is the random effect of the tray the plant was grown in, and *𝑒*_rwt_ is the error term. BLUPs were extracted using R/lme4::ranef.

We used procrustes analysis to determine concordance between all predicted RF anatomical traits and observed BLUPs. The procrustes analysis relates the overall shape of two sets of multivariate matrices by minimizing the total distance between the two distributions and quantifying how much the relationship between variables in the matrices differs after this alignment (Goodall, 1991). The algorithm was implemented using the function R/vegan::procrustes (Oksanen et al., 2022).

### 2.10 Genome‐wide association in the Wisconsin Diversity Panel

We used genetic and phenotypic data for 175 inbred maize lines from the expanded Wisconsin Diversity Panel (WIDP; Schneider et al., 2020). We note that the WIDP data was collected from axial roots of node four while our RF models were trained using anatomical parameters of axial roots from node two. We filtered published SNP data for minor allele frequency >5%, resulting in a total of 370,991 SNPs. Thirteen root anatomical traits were extracted from the published study: RXSA, TCA, TSA, TSA.RXSA, TSA.TCA, AA, X.A, CCS, MA, MD, MVA, NMV, and CCFN. The full design and experimental protocol are described in Schneider et al. (2020). Briefly, maize genotypes were grown in two replicates under well‐watered and water‐stressed conditions in a randomized complete block design. Plots were irrigated with a center pivot system, and water stress was applied 4 weeks after planting. At anthesis, one representative plant from each plot was excavated from the soil using a standard shovel. Root crowns were soaked and washed to remove soil particles. A representative fourth node root (5–8 cm from the base of the root) was excised imaged and phenotyped for root anatomical traits using LAT and *RootScan* imaging software (Burton et al., 2012). We fitted a linear mixed effect model using R/lme4 (Bates et al., 2007) for the well‐watered and water‐stressed condition with overall mean as the fixed effect and genotype and block as random effects and extracted BLUPs for genotypes with the R/lme4::ranef function. Broad sense heritability for each root trait was estimated as the genotype variance divided by the sum of genotype variance and error variance from linear mixed effect models. Root trait BLUPs were used to fit linear mixed models using R/GridLMM, a package for fitting linear mixed models with multiple random effects (Runcie & Crawford, 2019). We used the function R/GridLMM::GridLMM_GWAS to run the GWA study and set the environmental vector to −1 or 1 in the model to represent the water‐stressed and well‐watered treatments. The *p*‐values for the genotype main effect and the genotype by environment interaction effect were calculated using Wald tests. The SNP level *p*‐values were combined into the gene level associations using Multi‐marker Analysis of GenoMic Annotation (MAGMA) (de Leeuw et al., 2015). MAGMA uses a multiple regression model to aggregate all SNP information for a gene while accounting for linkage disequilibrium (LD). SNPs were annotated to genes using a 2.5 kb window around each gene, resulting in 24,099 genes.

### 2.11 Environmental GWA in the CIMMyT panel

We used MAGMA (de Leeuw et al., 2015) to estimate gene‐level associations between CIMMyT genotypes and environmental descriptorss. The first five eigenvectors of the genetic relationship matrix were included in the model to control for population structure. SNPs were annotated to genes using a 2.5 kb window around each gene. The final dataset contained 1656 genotypes and 28,898 genes for CIMMyT panel accessions.

### 2.12 Common genes captured in both the WIDP and CIMMyT panels

After gene annotation, we obtained 21,883 genes that were captured by associated SNPs in both the WIDP panel and CIMMyT panel maker sets. To evaluate if genes that are highly associated with root anatomical traits also showed associations with our predicted root traits and environmental variables, we extracted and pooled candidate genes from the top 100 genes for all WIDP root traits, predicted root traits, and related environmental variables identified by MAGMA. The final gene list contained WIDP root anatomical genes (576 genotype main effect genes and 542 WIDP genotype × treatment interaction genes), 636 RF predicted anatomical genes, and 1282 environmental genes (Supporting Files in Data S1).

### 2.13 Minor allele frequency in CIMMyT root clusters

To characterize the relationship between allele variation, environment, and root traits, we calculated the mean elevation, the minor allele frequencies (MAF) of the candidate SNPs from GWA, and the mean predicted root traits for each CIMMyT root anatomy cluster. Pearson correlation was used to test the correlations between MAF and elevation, and between MAF and predicted root traits.

## 3 RESULTS

### 3.1 Root anatomy varies among Mexican native maize varieties

**FIGURE 1:**
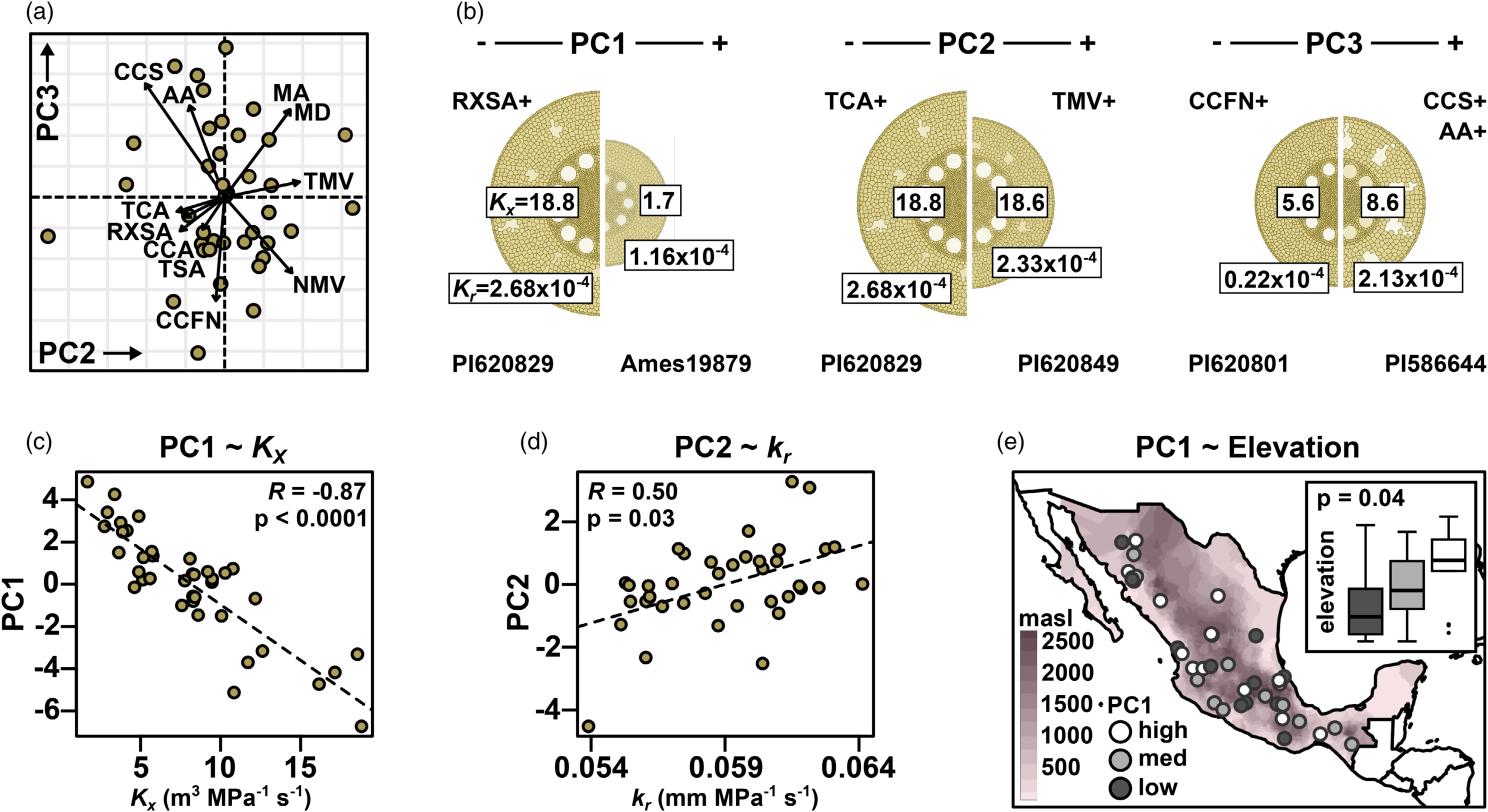
Root anatomy varies in Mexican native maize. (a) PC2 and PC3 loadings for root anatomical traits of accessions from Burton et al. (2013). Trait description and codes as Table 1. (b) Representative cross sections of extremely high‐ and low‐loading individuals for PCs 1–3 rendered using GRANAR, scaled to the measured *root cross‐section area*. Trait codes indicate broad trends seen in trait loading on the PCs. Boxed numbers adjacent to the central stele show modeled axial conductance (*K*_x_). Boxed numbers on the outer epidermis show modeled radial conductance (*K*_r_). Accession numbers are given at the base of the images. (c) Correlation between modeled *K*_x_ and anatomical PC1. (d) Correlation between modeled radial conductivity (*k*_r_) and anatomical PC2. (e) Accession source labeled by loadings on PC1, divided into terciles as low, medium (med) or high. Base map shaded by elevation. Inset box plots show the median and quartile elevation for the low, med and high PC loading groups. Whiskers extend to the most extreme points within 1.5× box length; outlying values beyond this range are shown as points. Stated *p*‐value refers to an ANOVA for differences in elevation among the PC1 tercile groups.

To characterize the relationship between root anatomy and environment in Mexican native maize, we assigned georeference data to 39 Mexican accessions phenotypically characterized in a previous root anatomy study (Burton et al., 2013; hereafter, the Burton panel) and extracted associated climate and soil descriptors from publicly available databases (see Section 2). We supplemented published trait data with a re‐analysis of the original cross‐sectional images to generate a final phenotypic dataset of 16 anatomical traits (Table 1). We performed a principal component (PC) analysis on the phenotypic data: PC1 was negatively correlated with root cross‐sectional area (Figure S1; Figure 1b). PC2 and PC3 captured allometric relationships among traits (Figure 1a,b): PC2 was associated with variation in the relative contribution of the stele to the total area, with *total metaxylem vessel area* and associated metaxylem traits loading antagonistically to *total cortical area* and *root cross‐section area*; PC3 captured an apparent trade‐off between *cortical cell file number* and *cortical cell size* along with variation in cortical *aerenchyma area*. In addition to grouping traits by PC analysis, we used a modeling pipeline linking the GRANAR and MECHA packages to simulate cross‐sectional anatomy (Heymans et al., 2020) and predict (i) radial conductivity (*k*_r_) and (ii) radial and axial conductance (*K*_r_, *K*_x_; Couvreur et al., 2018; Figure S2). Axial and radial hydraulic conductance were negatively correlated with PC1, indicating the greater capacity of larger diameter roots for water transport (Figure 1c). PC1 and PC2 were both positively correlated with radial conductivity (Figure 1d), reflecting the greater ease of water transport across roots with less cortex (Heymans et al., 2020). Further associations between anatomical trait PCs and derived hydraulic properties were not easily captured by simple correlations (Figure S2).

**TABLE 1:** Description of root anatomical traits used in this study.

Abbreviation	Trait description
RXSA	Root cross‐section area (mm^2^)
TCA	Total cortical area (mm^2^)
TSA	Total stele area (mm^2^)
TSA.RXSA	Total stele area.root cross‐section area
TSA.TCA	Total stele area.total cortical area
AA	Aerenchyma area (mm^2^)
X.A	Percent of cortex as aerenchyma
CCA	Cortical cell area (mm^2^)
CCS	Cortical cell size (mm^2^)
X.CCA	Percent living cortical area
MA	Individual metaxylem vessel area (mm^2^)
MD	Individual metaxylem vessel diameter (mm)
MVA	Total metaxylem vessel area (mm^2^)
NMV	Number of metaxylem vessels
CCN	Number of cortical cells
CCFN	Cortical cell file number

As a first attempt to identify clinal relationships between root anatomy and local environment, we examined the correlation of root anatomy PCs to four basic environmental descriptors (elevation, annual precipitation, mean temperature, and soil pH). After adjusting p‐values for multiple comparison testing, we did not find any significant correlation for either root anatomy (Figures S3 and S4) or derived hydraulic properties (Figure S5), although there was an indication that PC1 was related to elevation (Figure S3). When dividing PC1 loadings into terciles, individuals in the upper tercile were, on average, sourced from the highest elevations (Figure 1e).

### 3.2 Combined environmental descriptors predict variation in root anatomy

**FIGURE 2:**
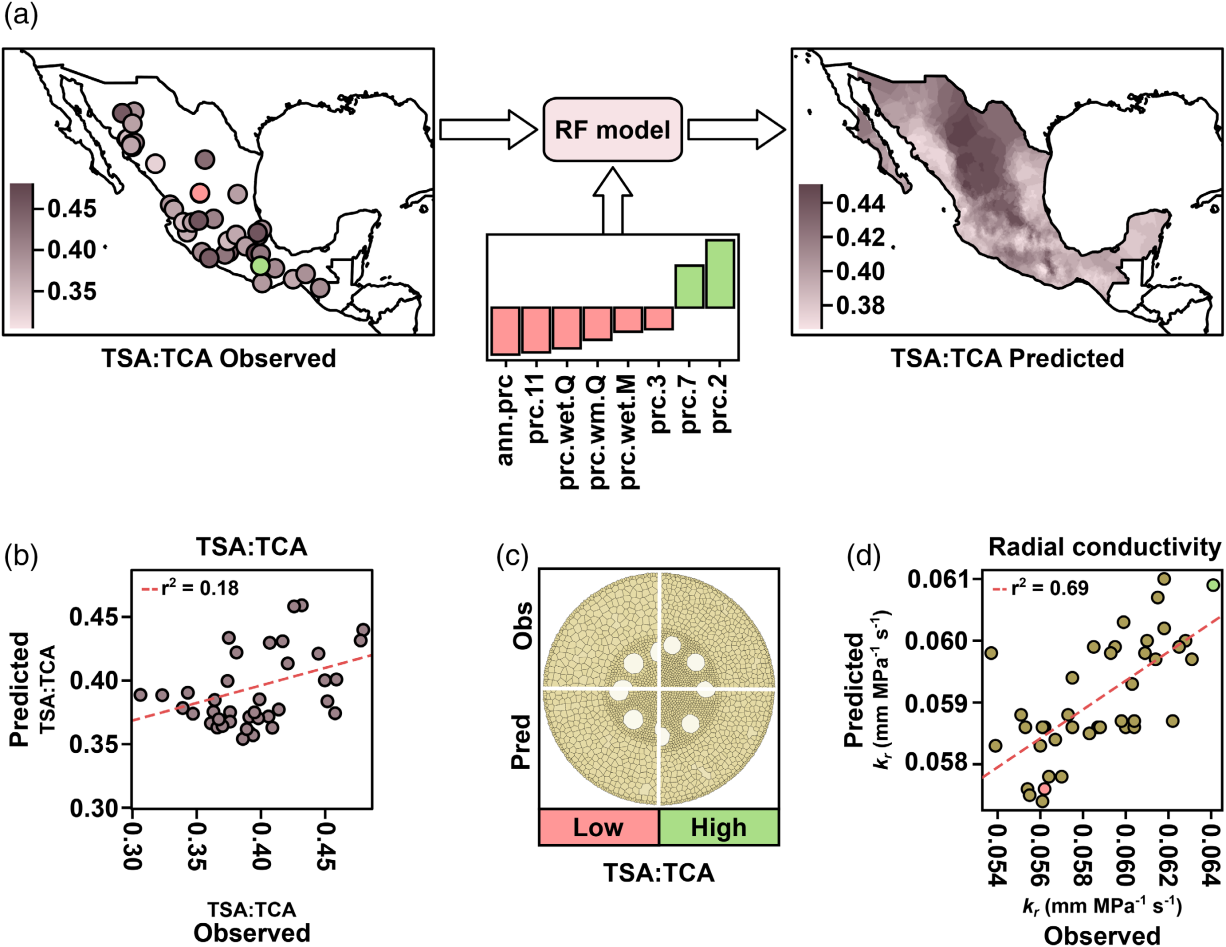
Home environment predicts root anatomy in Mexican native maize. (a) Random forest (RF) modeling for the ratio of *total stele area*:*total cortical area* (TSA:TCA). Accession point of origin colored by observed TSA:TCA from Burton et al. (2013). Individuals colored pink and green denote the accessions with the lowest (AMES19907) and highest (PI629263) observed TSA:TCA, respectively. Trait‐specific significant environmental descriptors identified by the Boruta method used for RF model construction, displayed as SHapley Additive exPlanations (SHAP) contributions. Smoothed RF predicted TSA:TCA for native Mexican maize. (b) RF predicted vs observed TSA:TCA values for all individuals used in model training and validation. (c) Composite GRANAR representation of observed (obs) and predicted (pred) GRANAR sections for the accessions with the lowest (low, pink) and highest (high, green) observed TSA:TCA. Predicted GRANAR cross‐sections use predictions for all traits for which RF models were constructed and are rendered at the same size. (d) Predicted versus observed radial conductivity (*k*_r_) for all individuals used in model training and validation. Predicted *k*_r_ was calculated using RF anatomical predictions and observed *k*_r_ was calculated using observed anatomical values from Burton et al. (2013). The individuals with the lowest and highest observed TSA:TCA are colored pink and green, respectively. Dashed line is the coefficient of determination for all plotted points.

Local adaptation is driven by varied aspects of the environment and their interactions. To capture more complex trait‐environment relationships, we used a feature‐reduction method (Boruta algorithm); to select the most informative of a full set of 157 available environmental descriptors for each anatomical trait, and subsequently combined the chosen descriptors into random forest (RF) models to relate environment and trait (Figure 2a; Figure S6). Nine of the 16 tested root anatomical traits were associated with environmental descriptors by the Boruta algorithm (Table S1). Thirty‐nine different environmental descriptors were used as input for RF models across the nine modeled anatomical traits, with individual models using from two (*percent of cortex as aerenchyma*) to sixteen (*total metaxylem vessel area*) environmental descriptors. We observed varying goodness‐of‐fit from RF models and the *R*‐squared for predicted versus observed trait values ranged from 0.32 (*aerenchyma area*) to 0.01 (*total metaxylem vessel area*) (Figure S7).

We used the GRANAR‐MECHA pipeline to combine predicted trait values and compared observed and predicted anatomies, both graphically (Figure 2c) and with respect to hydraulic properties (Figure 2d). Modeled conductivity and conductance values for predicted anatomies correlated well with values from the observed data (radial conductivity, *r* = 0.69, *p* < 0.01; radial conductance *r* = 0.28, *p* = 0.07; axial conductance *r* = 0.59, *p* < 0.01; Figure S8), indicating that our RF models successfully captured differences in anatomical traits that impact root hydraulic properties.

### 3.3 Random forest prediction of root anatomy across Mexican native maize

To estimate root anatomical diversity across a broader sampling of native Mexican maize, we applied our Burton‐trained RF models to a panel of 1791 genotyped and georeferenced Mexican accessions (hereafter, the CIMMyT panel; Romero Navarro et al., 2017; Figure S9). We used the georeference data to link environmental descriptors to each accession and passed these to the RF models, generating a complete phenotypic set of nine estimated root anatomical traits for the 1791 accessions (Supplementary Information). To summarize patterns among the predicted trait values, we used partition‐against‐medians (PAM) clustering (Klein et al., 2020; Maechler et al., 2021) to group the accessions into seven phenotypic clusters (Figures S10 and S11). The clusters 1 through 7 were composed of 308, 366, 370, 231, 158, 277, and 131 accessions, respectively. The structure defined by the clustering was not strong (mean silhouette value = 0.25), reflecting the continuous nature of the environmental descriptors driving the RF models, but did provide a context for subsequent analyses. We used the median trait values of each cluster to obtain average anatomies and hydraulic properties with the GRANAR‐MECHA pipeline.

**FIGURE 3:**
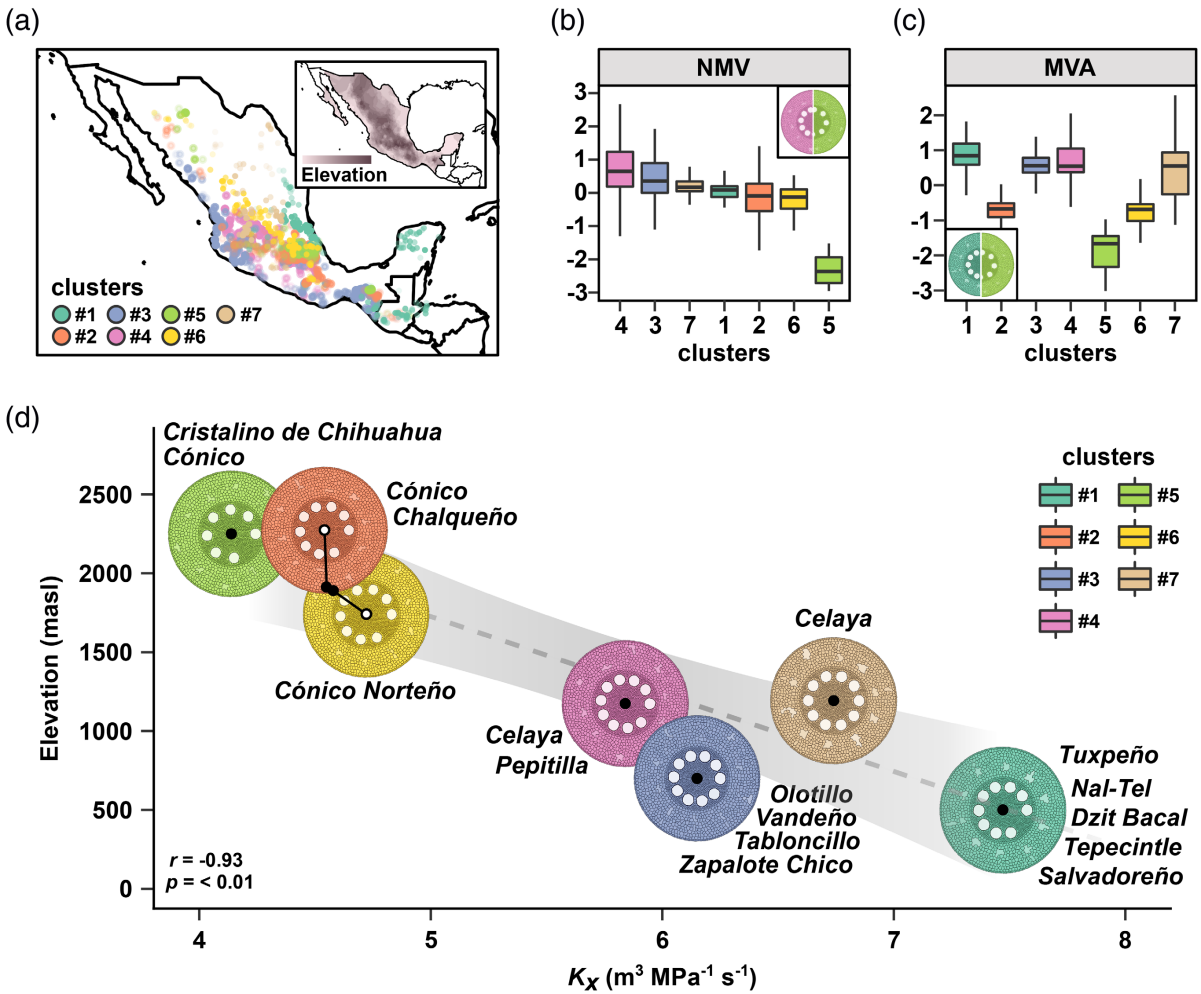
Groups defined by shared root anatomical characteristics originate from distinct environments. In this study, 1791 maize accessions forming the CIMMyT panel were grouped into seven clusters based on eight RF‐predicted root anatomical traits. (a) Geographical distribution of the clusters. Inset shows elevation, with darker shading corresponding to higher values. (b) Centered and scaled *Number of metaxylem vessels* (NMV) and (c) *Total metaxylem vessel area* (MVA) in the seven clusters. Inset shows composite GRANAR representation generated from the median trait values of highest (left) and lowest (right) scoring clusters. (d) Predicted axial conductance is negatively correlated with elevation. Black points indicate the mean axial conductance and elevation for each of the seven clusters. The gray dashed line is the regression fit with the shaded band showing 95% confidence. Pearson correlation and p value are shown in the bottom left. Root cross‐section images were generated with GRANAR using the median trait values for each cluster, all rendered at the same size. For clusters 2 and 6, root‐cross sections are plotted off the regression line for clarity. Varieties overrepresented in each cluster are listed next to the cross‐sections.

Clusters were distinguished by the relative elaboration of cortex and stele and associated hydraulic properties (Figure 3). In clusters 1 and 3, the stele (*total stele area*: *root cross‐section area*; *total stele area*: *total cortical area*) was relatively small, although individual metaxylem vessels were large (*individual metaxylem area*, *individual metaxylem diameter*) and, consequently, the *total metaxylem vessel area* and axial conductance were relatively high. In contrast, clusters 5, 2, and 6 were distinguished by a small stele and small metaxylem vessels, associated with low axial conductance relative to other clusters. In cluster 5, the small size of the metaxylem vessels was further associated with a low *number of metaxylem vessels* resulting in the lowest *total metaxylem vessel area* and the lowest axial conductance of the clusters.

We examined the clusters with respect to previous morphological‐isozymatic (Sanchez et al., 2000) and environmental (Ruiz Corral et al., 2008) classifications of Mexican native maize (Tables S2 and S3; Figure S11). Following trends reported for overall Mexican maize diversity (Sanchez et al., 2000), our clusters were structured with respect to elevation (Figure 3). Clusters 2, 5, and 6 were enriched (Fisher test for variety count in or out of the cluster, *p* < 0.05) for varieties belonging to the previously defined Highland group (Sanchez et al., 2000; Figure 3; Table S3). Cluster 5, containing the highest elevation varieties, was centered on Mexico City, although it also contained accessions from the highlands of Chihuahua in northern Mexico; cluster 6 extended from north to south along the Sierra Madre Occidental; cluster 2 was again centered on Mexico City, although with greater representation further west along the trans Mexican volcanic belt than cluster 6 and included several accessions from the Chiapas highlands on the southern border of Mexico. The mid‐elevation clusters 4 and 7 were loosely sourced from the center‐to‐south and center‐to‐north of Mexico, respectively. Clusters 1 and 3 were enriched for varieties in the Lowland Short‐to‐Medium Maturity and Tropical Dent groups (Sanchez et al., 2000; Tables S2 and S3), with cluster 1 from the Gulf Coast, the Yucatan and lowland Guatemala and cluster 3 from the Pacific Coast. The prior environmental classification was in line with the observed elevational cline: clusters 2, 5, and 6 were enriched for varieties previously assigned to *Temperate to Semi‐Hot* environments; clusters 1 and 3 were enriched for varieties assigned to the *Very Hot* niche (Ruiz Corral et al., 2008; Figure S12, Table S3).

Considering the average predicted anatomies and mean values of environmental descriptors associated with each cluster, we could discern a significant trend of reduced axial conductance with increasing elevation (Figure 3d). Our models associated the colder, drier highland niche (>2500 masl; Eagles & Lothrop, 1994; Ruiz Corral et al., 2008) with both fewer and smaller metaxylem vessel elements (cluster 5). Conversely, the hot, wet lowlands (Ruiz Corral et al., 2008) were associated with a relatively larger stele accommodating a greater number of larger metaxylem vessel elements (clusters 1 and 3).

### 3.4 Phenotypic evaluation of previously uncharacterized varieties supports RF‐predicted variation in axial conductance

**FIGURE 4:**
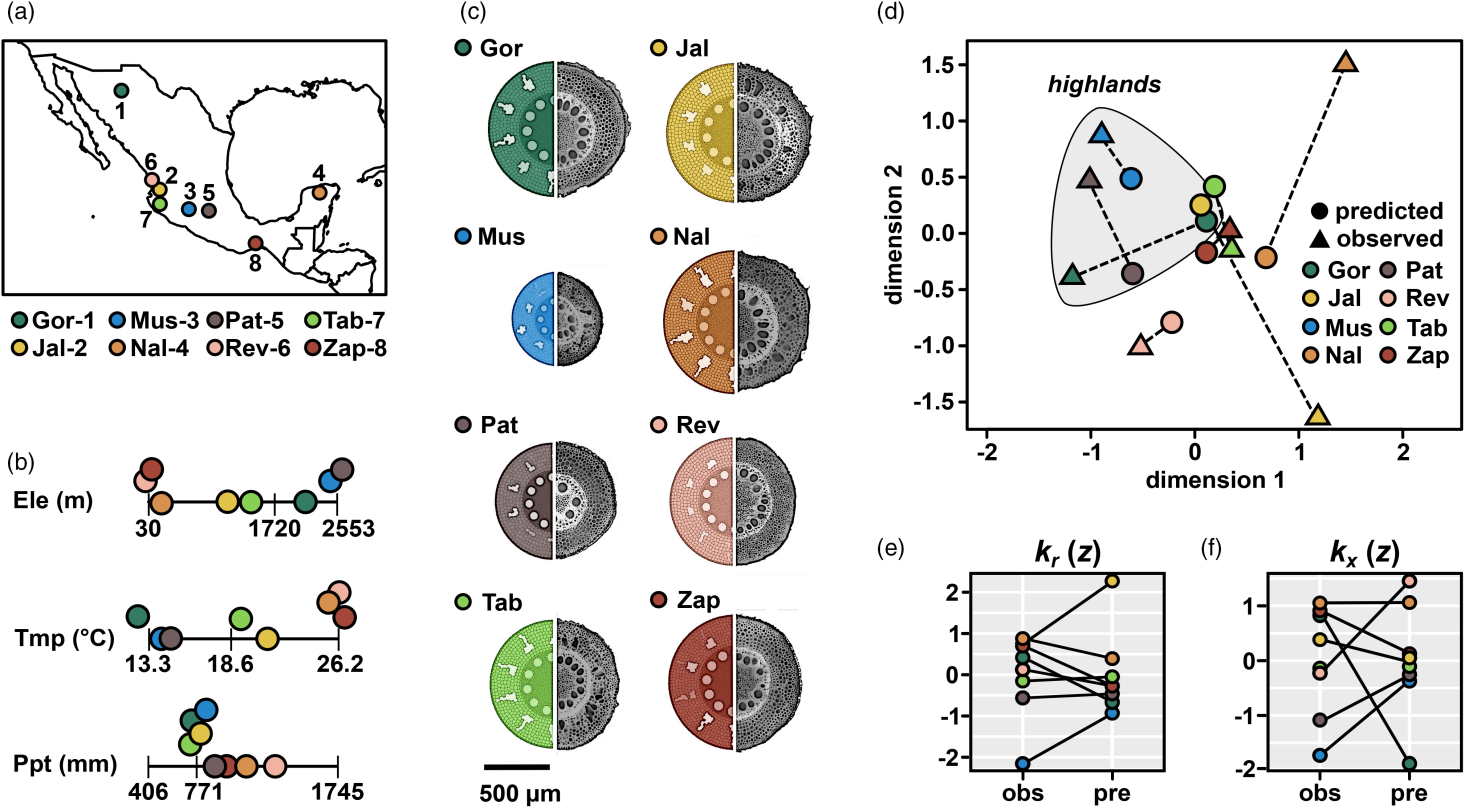
Novel phenotypic data is consistent with model predictions. (a) Source of eight selected native maize varieties: (1) Gordo, (2) Jala, (3) Mushito, (4) Nal Tel, (5) Palomero Toluqueño, (6) Reventador, (7) Tabloncillo, (8) Zapalote Chico. (b) Elevation (Ele), annual mean temperature (Tmp), and annual precipitation (Ppt) at source locality for the eight native varieties. Bars on the line plots represent the 5%, 50%, and 95% quantiles for each environmental descriptor across all CIMMyT panel individuals included in this study (1791). Points color‐coded as (a). (c) GRANAR renderings of observed anatomical BLUPs across node two and three roots (colored) and photographs of representative cross‐sections of third‐node roots, scaled to the mean measured *root cross‐section area*. (d) Procrustes analysis comparing the distribution of RF‐predicted (circles) and observed (triangles) anatomical traits. For each variety, predicted and observed projections are linked with a dotted line. Shading was added to highlight three varieties sourced from high elevation. (e) Comparison of standardized observed (obs) and predicted (pre) values of modeled radial conductance (*K*_r_). Values for each variety are connected to illustrate the level of consistency in ranking. (f) as (e), showing modeled axial conductance (*K*_x_).

To empirically evaluate our RF models, we characterized eight maize accessions from across Mexico that had not been used previously in the Burton study (Figure 4a,b). We measured root anatomical traits following the Burton protocol, and for each trait compared the observed best linear unbiased predictor (BLUP) with the results of our environmental RF predictions (Figure S13). The correlation between observed BLUPs and RF predictions ranged from relatively high for cortical traits (*aerenchyma area*, *r* = 0.71; *percent of cortex as aerenchyma*, *r* = 0.65; *percent of cortex as cortical cells*, *r* = 0.52) to lower for metaxylem vessel traits (*number of metaxylem vessels*, *r* = 0.30; *total metaxylem vessel area*, *r* = 0.27; *individual metaxylem vessel area*, *r* = 0.11) and allometric traits (*total stele area*:*root cross‐section area*, *r* = 0.18; *total stele area*:*total cortical area r* = 0.06). Predictions of *individual metaxylem vessel diameter* were not well supported by observed values (*r* = −0.24).

We assessed overall concordance between observed and predicted anatomy by using the Procrustes transformation (Schönemann, 1966) to minimize the distance between each set of observed and predicted trait values across the eight accessions. Observed and predicted root anatomy was well‐matched for six of the eight accessions (Figure 4d; Figure S14). The difference in overall root anatomy in varieties sourced from the highlands and lowlands was well supported by both observed and predicted trait values (Figure 4d; Figure S15) and modeled hydraulic properties (Figure 4e,f). Overall, although individual traits were not always well predicted for any given accession, our methodology and training data were sufficient to capture a broad stratification of anatomical traits and hydraulic properties across the environment.

### 3.5 Genome‐wide association analysis using predicted trait values identifies novel candidate genes

To characterize the genetic evidence linking root anatomy to the local environment in Mexican maize, we ran a GWA analysis on the CIMMyT panel using the RF‐predicted trait values. Given the nature of the RF models, this prediction GWA is, in effect, a development of a standard environmental GWA analysis with modeled trait values capturing complex combinations of the individual environmental descriptors used in RF model construction. For comparison, we ran separate environmental GWA analyses for each of the 39 environmental descriptors used in RF modeling and also re‐analyzed the most extensive maize root anatomy data set available, a published study including 175 modern maize inbred lines grown in well‐watered and water‐limited treatments (hereafter, the WIDP panel). For the WIDP panel, we extracted phenotypic data for eight of our nine RF modeled traits (not including *percent of living cortical area*), combining values obtained for well‐watered and water‐limited treatments into a single GWA model, estimating variant main (G) and variant x treatment (GxE) effects. To facilitate comparison across panels genotyped using different platforms, we used the MAGMA pipeline to combine signals across single nucleotide polymorphisms (SNPs) to a single gene level value. Here, we assigned any SNP ± 2.5 kb from an annotated gene model to that gene. In the following discussion of overlap between our different GWA analyses, we consider only genes captured in both CIMMyT and WIDP markersets. In later identification of the genes of greatest interest from the predicted GWA analysis, we do not take the WIDP markeset into account.

**FIGURE 5:**
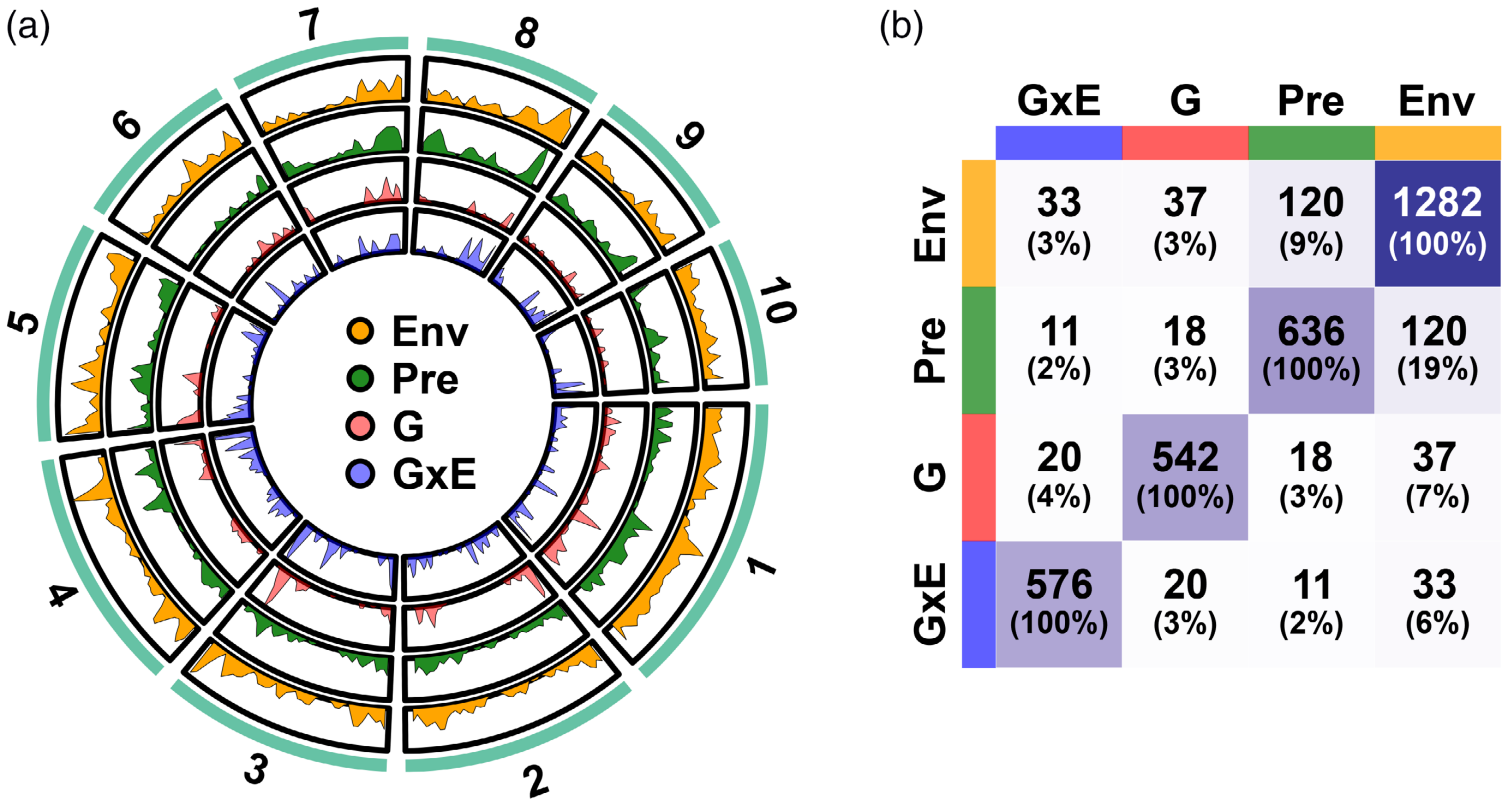
Evidence for a shared genetic basis of root anatomical variation between inbred breeding lines and Mexican native maize. (a) Distribution of top 100 genes from GWA analyses of WIDP and CIMMyT accessions across the genome. The shaded area represents the density of top genes overlapped with window regions (1 × 10^7^ bp). Sector names represent the number of chromosomes. (b) The number of pairwise overlapping genes among the GWA gene sets. The darker the color of the squares indicates a higher number of genes. Totals in parentheses show the percentage of the row set in the other sets.

To compare CIMMyT “predicted,” CIMMyT “environment,” WIDP “G,” and WIDP “GxE” GWA analyses, we selected the top 100 genes (determined by *p*‐value) per trait for each analysis and combined these into candidate gene lists, obtaining sets of 636 unique prediction genes, 1282 unique environment genes, 542 unique G genes, and 576 unique GxE genes (Figure 5; Supplementary Information). Only 19% of the prediction genes were also present in the environment set (Figure 5b), indicating that the two were not redundant and that the prediction set was capturing patterns not revealed by separate analyses of the individual environmental descriptors. For example, a region of the short arm of chromosome 10 was linked to *mean metaxylem vessel diameter* in the prediction analysis (Figure S16). In this case, the −log_10_(*p*) value of the most significant SNP for the predicted trait is approximately double that of the best‐supported environmental descriptor (precipitation in October). The best‐supported SNP in this region fell within the gene *Trichome birefringence‐like 10* (*Tbl10*; Zm00001d023378; Figure S16). Natural variation in *Tbl10* has previously been linked to variation in flowering time (Chen et al., 2012; Kusmec et al., 2017), height (Wang et al., 2023), and root diameter (Pace et al., 2015). As such, *Tbl10* illustrates a compelling candidate for further follow‐up that would not have been identified by standard environmental GWA.

### 3.6 The gene *Vq29* is linked to variation in both metaxylem traits and source elevation

**FIGURE 6:**
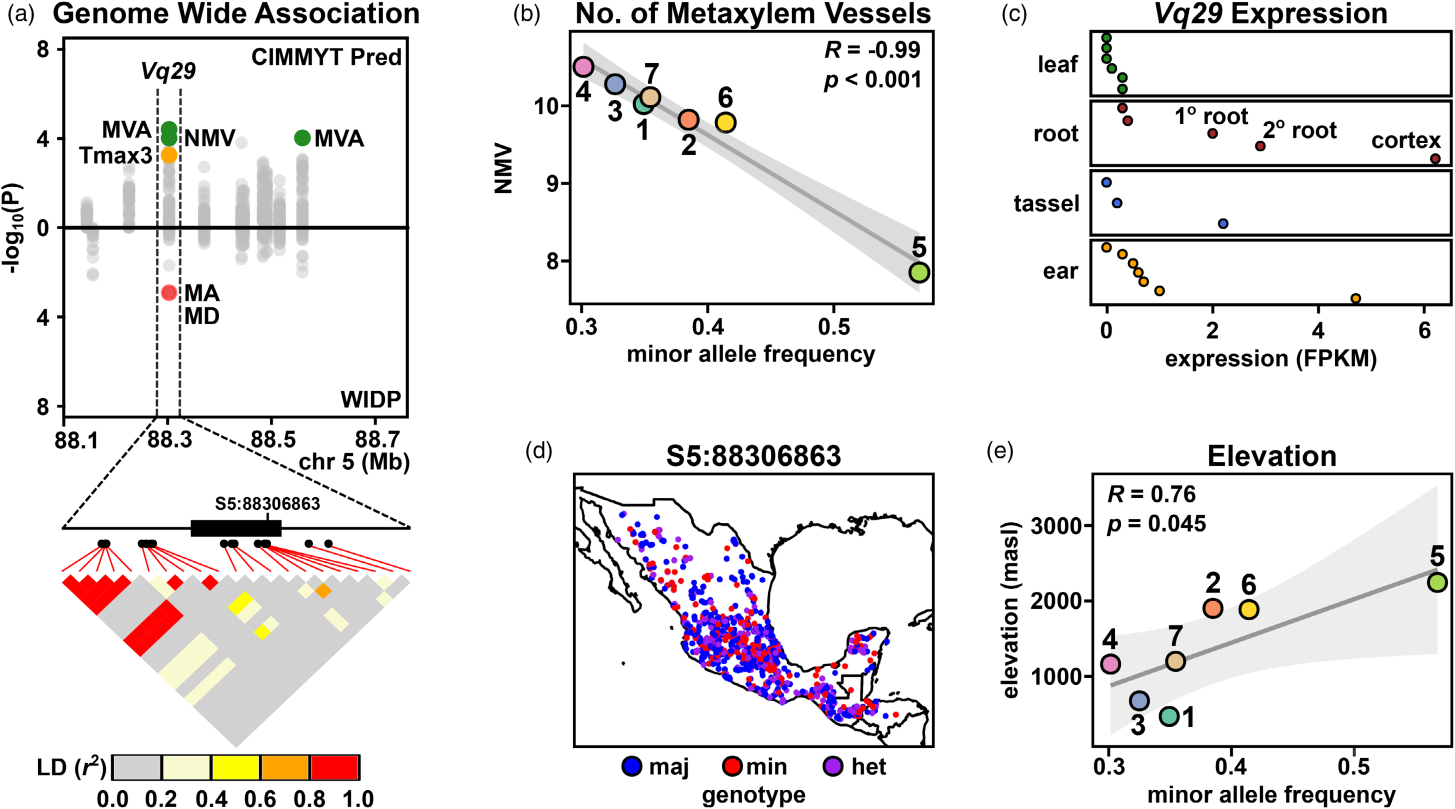
The gene *Vq29* is linked to variation in root anatomy and source environment. (a) Miami plot showing GWA support (−log_10_ *p*) for association with root anatomy for genes on a region of chromosome 5. Points above the *x*‐axis show support for predicted phenotypes in the CIMMyT panel; points below the *x*‐axis show support for observed phenotypes for the WIDP panel. The gene *Vq29* is associated with *total metaxylem vessel area* (MVA), *number of metaxylem vessels* (NMV), *individual metaxylem vessel area* (MA), and *individual metaxylem vessel diameter* (MD) across the two analyses. Image below the Miami plot shows the *Vq29* gene model (CDS as filled box), SNP position (filled circles), and pairwise linkage disequilibrium (LD). The position of the focal SNP S5:88306863 is highlighted. (b) Correlation between frequency of the minor allele at S5:88306863 in the previously defined CIMMyT clusters and mean predicted NMV. (c) Expression of *Vq29* in four named tissues from publicly available expression data. Points show different subsamples. The root cortex, corresponding to the highest expression, is highlighted. (d) Geographic allele distribution of S5:88306863 in the CIMMyT panel. (e) Correlation between frequency of the minor allele at S5:88306863 and mean elevation at source in the CIMMyT clusters.

There was no evidence that the prediction set was enriched for WIDP root anatomy candidate genes with respect to the environment set; both contained 2%–3% WIDP G and GxE genes (Figure 5b). Nonetheless, we consider the 29 genes identified in both prediction and WIDP (G and/or GxE) GWA to be high‐confidence candidates for further characterization (Figure 5b; Figure S17, Table S4). For example, the gene *Vq29* (Zm00001d015397) on the short arm of chromosome 5 was associated with *number of metaxylem vessels* and *total metaxylem vessel area* in the prediction GWA and with *individual metaxylem vessel area* and *individual metaxylem vessel diameter* in the WIDP G analysis (Figure 6a). The *Vq29* gene is predicted to encode a VQ domain transcription factor, part of a large family of proteins that interact with members of the WRKY family under stress (Song et al., 2015), including in response to hypoxia, ozone, or nitric oxide (León et al., 2020). The minor allele of the highest scoring SNP (S5:88306863) was associated with lower *number of metaxylem vessels* and *total metaxylem vessel area* and declined in frequency within our clusters with increasing predicted values of these same traits (Figure 6b). Based on gene expression atlas data (Walley et al., 2016), *Vq29* is most highly expressed in the roots, consistent with a role in metaxylem development (Figure 6c). Geographically, the minor allele of S5:88306863 was most prevalent in the central Mexican highlands (Figure 6d), and the MAF increased with mean elevation across our previously defined clusters (Figure 6e). In summary, *Vq29* nicely illustrates an example of a candidate gene associated with phenotypic variation in root anatomy in the inbred WIDP panel that also shows clinal genetic variation across the Mexican environment.

## 4 DISCUSSION

We have presented evidence that variation in root anatomy may contribute to local adaptation in Mexican native maize. We used predictive models to define biologically relevant clines over which we identified both genotypic and phenotypic variation. Shared GWA candidates between Mexican native maize and modern inbred lines indicated an element of common genetic architecture, although we also identified novel candidates specific to the native Mexican material. Phenotypic patterns suggested that local differences in precipitation and temperature are associated with heritable variation in maize root anatomy. Root anatomical variation broadly followed the established grouping of Mexican maize varieties, themselves strongly stratified by environment. Our observations are consistent with a role for root anatomical variation in local adaptation. Our combination of environment‐based models and GWA allowed us to leverage a relatively small sample of phenotypically characterized locally adapted varieties to identify novel associations between phenotype, genotype, and environment in the context of broader maize diversity.

Our analyses highlight a predominance of anatomies predicted to reduce axial conductance in varieties sourced from arid subtropical or temperate environments. In both observed and predicted phenotypic data, varieties from the cooler, drier highland regions were associated with fewer and/or narrower metaxylem vessels and a reduction in the stele area with respect to cortex. Comprehensive revision of data across taxa has previously suggested that the capacity for axial water transport is typically greater in plants from wet environments and reduced in plants adapted to xeric conditions (Feng et al., 2016; Lynch et al., 2021). Although somewhat counterintuitive, reducing water uptake under dry conditions may benefit plants by reducing root tip desiccation (Richards & Passioura, 1989), preventing cavitation (Nardini et al., 2013), and enabling the conservation of soil water resources across the growing season (Leitner et al., 2014; Richards & Passioura, 1989). Studies of interspecific variation in crops support these hypotheses with both narrower metaxylem vessels (Allah et al., 2010; Peña‐Valdivia et al. 2005; Priatama et al., 2022; Purushothaman et al., 2013) and fewer metaxylem vessels (Strock et al., 2021) being associated with enhanced drought tolerance. Similarly, selection for reduced xylem vessel diameter in Australian wheat has been reported to successfully increase yield under water limitation (Richards & Passioura, 1989). In the Mexican highlands, farmers traditionally plant prior to the beginning of the annual rains to maximize the length of the growing season and ensure crops reach maturity prior to the first frosts (Eagles & Lothrop, 1994). As a consequence, seed is deep planted to better access residual soil moisture, as well as to offer protection from low temperatures, a practice also employed in the southwestern US (Collins, 1914). For wheat varieties reliant on residual soil moisture during early growth, reduced root conductance has been correlated with increased yield (Passioura, 1972). Mexican highland maize may similarly benefit from rationing water use early in the season (Fischer et al., 1983; Hayano‐Kanashiro et al., 2009) with reduced axial conductance contributing to this adaptive water‐saving strategy.

We observed variation in the formation of root cortical aerenchyma, with varieties sourced from regions of higher precipitation generally being associated with greater aerenchyma formation. Root cortical aerenchyma forms constitutively in wetland crops such as rice and in maize wild relatives endemic to regions of high precipitation (Mano et al., 2007; Mano & Nakazono, 2021). Many cultivated maize genotypes lack constitutive aerenchyma; however, aerenchyma formation can be induced by environmental stresses, such as hypoxia (Yamauchi et al., 2016), drought (Zhu et al., 2010), heat (Hu et al., 2014) or nutrient starvation (Galindo‐Castañeda et al., 2018; Saengwilai et al., 2014). Although greenhouse evaluation was conducted in benign conditions, substantial aerenchyma production was observed (11% Jala, this study; 16% PI586644 in Burton et al., 2013). In the field, aerenchyma plays a role in oxygenation of the root tissue under hypoxia (Colmer, 2003; Jackson et al., 1985), while, in resource‐limited conditions, the reduction in root metabolic cost resulting from aerenchyma formation may enhance the efficiency of foraging in terms of carbon invested (Klein et al., 2020; Lynch et al., 2021). On the other hand, with fewer living cortical cells a plant may be less able to accommodate mutualistic arbuscular mycorrhizal fungi, although the relationships between root anatomy, microbial interactions, environment, and cortical burden remain to be fully understood (Galindo‐Castañeda et al., 2018; Saengwilai et al., 2014; Strock et al., 2019). In our predictive analysis high *aerenchyma area* was associated with the varieties from the Gulf Coast, the Yucatan (exemplified by Nal Tel in our greenhouse evaluation), and the region around the southern Mexican border extending into Guatemala. This last region covers the native range of the flooding tolerant teosinte *Zea mays* ssp. *huehuetenangensis* (Mano et al., 2005) and suggests flooding may have also exerted selective pressure on endemic maize.

The functional impact of root anatomical variation is contingent on root system architecture and, indeed, overall plant phenology (Lynch, 2019). Differences in growth angle and branching determine the deployment of roots across the soil profile and will interact, synergistically or antagonistically, with root anatomy to impact overall root function. For example, the water‐banking effect of reduced axial conductance discussed above has been shown to be enhanced in the context of a shallow root system architecture in bean, enhancing plant performance under drought (Strock et al., 2021). While there is a scarcity of information concerning root system architecture in Mexican maize, the limited data reveal remarkable structural diversity, indicating strong spatio‐temporal variation in soil exploration (Heymans, 2022). It has been noted that native maize root systems tend to be generally shallower compared to those of inbred lines (Burton et al., 2013; Ren et al., 2022). Interestingly, the highland varieties we found associated with reduced axial conductance have previously been described to have a high tendency to lodge (fall over) due to “poorly developed” root systems (Wellhausen et al., 1952). In practice, traditional management involves pilling of earth around the growing plant, freeing the root system from the need to provide mechanical support and perhaps allowing an overall reduction in root system development that contributes to water‐banking.

In summary, our analyses indicate that reported variation in Mexican native maize root anatomy is distributed systematically over the environment, consistent with a role in local adaptation. We propose that predictive models based on a set of “signpost” accessions can define biologically relevant clines though complex environments, providing the appropriate axes against which to identify both phenotypic and genetic trends. Significantly, we obtained candidate genes from our predicted trait GWA that were not identified in one‐by‐one analyses of environmental descriptors, including candidates whose functional role in root anatomy is supported by previous studies of inbred maize (Figure 5; Klein et al., 2020; Schneider et al., 2020). The combined use of field evaluation and in silico modeling has allowed great progress to be made in defining the functional impact of root anatomical variation (Heymans et al., 2020; Lynch et al., 2021; Sidhu et al., 2023). The further study of locally adapted native varieties has the potential to complement these other approaches. The history of native crop diversity is a natural experiment that has run for thousands of years, selection imposed by environmental conditions being integrated over many generations. As such, subtle signals that can be hard to detect in experimental evaluation may be amplified and detected as patterns of GEA.

## FUNDING INFORMATION

This work was supported by Penn State SNIP2 project Root system functionality in cereals and USDA‐NIFA 2022‐67013‐37038 Project Functional Characterization of Maize Nitrogen Use Efficiency QTL and their Role in Nitrogen‐Phosphorus Signaling. RJHS is funded by USDA Hatch Appropriations under Project #PEN04734 and Accession #1021929.

## CONFLICT OF INTEREST STATEMENT

The authors declare no conflict of interest.

## Supporting information


Data S1.

Data S2.

## Data Availability

The data and scripts that support the findings of this study are available in a GitHub repository: https://github.com/chloee‐mclaughlin/MaizeRootDiversity (doi: 10.5281/zenodo.10631283).
