# Supplementary material for: Evidence that variation in root anatomy contributes to local adaptation in Mexican native maize
Source: Evol Appl. 2024 Mar 10;17(3):e13673. doi: 10.1111/eva.13673 (PMC10925829; doi:10.1111/eva.13673)
Supplement: Supplementary file 2 — Data S2. [file EVA-17-e13673-s001.pdf]

Supplemental Table 1 Anatomical traits random forest model were constructed for, the Boruta-identified environmental descriptors used in model construction, and the model percent variation explained. Adjusted R-squared (corrected goodness-of-fit) and p-values are from a linear model comparing predicted random forest trait values and observed trait values reported in Burton et al., 2013.

**Phenotype**	**Boruta-identified environmental descriptors**	**RF model percent variation explained**	**Adjusted R-squared**	**P-value**
TSA.RXSA	prec_3, prec_7, prec_10, prec_11, Ann.Prc, Prc.Wrm.Q	14.35%	0.15	0.0079
TSA.TCA	prec_2, prec_3, prec_7, prec_11, Ann.Prc, Prc.Wet.M, Prc.Wet.Q, Prc.Wrm.Q	16.69%	0.18	0.0046
AA (mm^2)	CACO3, BS, PARWin	33.32%	0.32	<0.0001
X.A (%)	BS, PARFall	20.28%	0.19	0.0029
X.CCA (%)	org_1m, tmin_4, tmin_10, pet_annual, pet_5, pet_6, pet_9	23.12%	0.21	0.0018
MA (mm^2)	coarse_1m, tmax_2, tmean_3, pet_4	23.05%	0.21	0.0019
MD (mm)	tmax_2, tmax_3, tmax_4, tmin_3, tmean_3, tmean_5, Ann.Mean.Tmp	2.12%	0.05	0.0958

NMV	tmax_10, tmax_11, tmin_3, tmin_6, tmin_8, tmean_5, tmean_10, tmean_11, Mean.Tmp.Dry.Q, pet_1, pet_12	9.46%	0.16	0.0294
MVA (mm^2)	tmax_1, tmax_2, tmax_3, tmax_4, tmax_11, tmax_12, tmin_3, tmin_4, tmin_5, tmean_2, tmean_3, tmean_4, tmean_5, tmean_11, pet_7, pet_8	-5.34	0.01	0.2349

Supplemental Table 2 For each CIMMyT PAM cluster assignment, mean elevation, modal assignment of morphological-isozymatic classifications (Ruiz Corral et al. 2008), modal assignment of environmental classifications (Sanchez G. et al. 2000), mean calculated radial conductivity, mean axial and radial conductance.

Cluster	Mean elevation (m)	Environmental classifications (Ruiz Corral et al. 2008)	Morphological-isozymatic classifications (Sanchez G. et al. 2000)	Radial conductivity (k_r) m MPa^{-1} s^{-1}	Axial conductance (K_x) m^3 MPa^{-1} s^{-1}	Radial conductance (K_r) m^3 MPa^{-1} s^{-1}
1	502	Very hot	Tropical dent	5.92 x 10^{-5}	7.47	2.06 x 10^{-4}
2	1912	Temperate to semi-hot	Highland	5.87 x 10^{-5}	4.55	2.05 x 10^{-4}
3	701	Very hot	Tropical dent	5.87 x 10^{-5}	6.15	2.05 x 10^{-4}
4	1175	Temperate to semi-hot	Tropical dent	5.99 x 10^{-5}	5.84	2.09 x 10^{-4}
5	2249	Temperate to semi-hot	Highland	5.87 x 10^{-5}	4.14	2.05 x 10^{-4}
6	1889	Temperate to semi-hot	Highland	6.09 x 10^{-5}	4.58	2.12 x 10^{-4}
7	1193	Temperate to semi-hot	Tropical dent	5.92 x 10^{-5}	6.74	2.06 x 10^{-4}

Supplemental Table 3 Enrichment of Mexican maize varieties in root anatomical clusters.

**Variety**	**Environmental classifications (Ruiz Corral et al. 2008)**	**Morphological-isozymatic classifications (Sanchez G. et al. 2000)**	**Cluster**	**Count variety in cluster**	**Count not variety in cluster**	**Count variety not in cluster**	**Count not variety not in cluster**	**Adjusted P-value**
Celaya	1b: Temperate to Semi-Hot	3b: Tropical Dent Group	4	33	159	79	1183	0.0010
Celaya	1b: Temperate to Semi-Hot	3b: Tropical Dent Group	7	24	81	88	1261	0.0001
Chalqueño	1b: Temperate to Semi-Hot	1a: Highland Cónico Group	2	39	260	67	1088	0.0170
Cónico	1b: Temperate to Semi-Hot	1a: Highland Cónico Group	2	109	190	151	1004	0.0000
Cónico	1b: Temperate to Semi-Hot	1a: Highland Cónico Group	5	60	59	200	1135	0.0000
Cónico Norteño	1b: Temperate to Semi-Hot	1a: Highland Cónico Group	6	43	155	54	1202	0.0000
Cristalino de Chihuahua	1a: Temperate to Semi-Hot, Semi-Dry	1b: Highland Chihuahua Group	5	9	110	9	1326	0.0010
Dzit Bacal	3: Very Hot	3a: Mid- to Highland Late Maturity Group	1	13	237	1	1203	0.0000

Nal Tel	3: Very Hot	3c: Lowland Short to Medium Maturity Group	1	25	225	16	1188	0.0000
Olotillo	3: Very Hot	3a: Mid- to Highland Late Maturity Group	3	24	267	15	1148	0.0000
Pepitilla	2c: Semi-Hot to Hot, Semi-Wet	3c: Lowland Short to Medium Maturity Group	4	18	174	23	1239	0.0003
Salvadoreñ o	N/A	N/A	1	15	235	1	1203	0.0000
Tepecintle	3: Very Hot	3c: Lowland Short to Medium Maturity Group	1	16	234	20	1184	0.0324
Tabloncillo	2c: Semi-Hot to Hot, Semi-Wet	2: 8-Row Group	3	25	266	29	1134	0.0039
Tuxpeño	3: Very Hot	3b: Tropical Dent Group	1	112	138	184	1020	0.0000
Vandeño	3: Very Hot	3b: Tropical Dent Group	3	19	272	16	1147	0.0018
Zapalote Chico	3: Very Hot	3c: Lowland Short to Medium Maturity Group	3	11	280	5	1158	0.0093

Supplementary Table 4 The gene location and annotation of top overlap genes between WIDP and Prediction GWA.

GENE	CHR	START	STOP	Predict	WIDP	Gene Name	Gene Symbol
**WIDP G and Prediction GWA**							
Zm00001d028214	1	26608063	26616524	MA	TSA.RXSA, TSA.TCA	.	.
Zm00001d028831	1	47987303	47994267	MA	NMV	.	.
Zm00001d030005	1	98582927	98597280	NMV	TSA.RXSA	.	.
Zm00001d033829	1	275155553	275162538	MD	AA	.	.
Zm00001d033830	1	275157506	275167900	MD	AA	.	.
Zm00001d002535	2	14917548	14923069	MA, MD, MVA	TSA.RXSA, TSA.TCA	.	.
Zm00001d006052	2	196573325	196584441	X.A	MA, MD	.	.
Zm00001d006053	2	196580004	196586817	X.A	MA, MD	NAC-transcription factor 24	nactf24
Zm00001d039901	3	18642659	18653125	MA	NMV	.	.
Zm00001d039902	3	18643645	18649520	MA, MD	NMV	.	.
Zm00001d054106	4	246772883	246783465	AA, X.A	AA, X.A	Protein disulfide isomerase3	pdi3
Zm00001d013067	5	4207061	4213378	X.A	X.A	.	.
Zm00001d015376	5	87049440	87058148	AA, X.A	MA, MD	Phosphoglycerate kinase3	pgk3

Zm00001d015397	5	88303760	88309464	MVA, NMV	MA, MD	VQ motif-transcription factor29	vq29
Zm00001d016842	5	178207500	178214684	TSA.RXSA, TSA.TCA	NMV	.	.
Zm00001d036241	6	78621119	78628756	MD	NMV	.	.
Zm00001d012089	8	168026879	168032905	MVA, NMV	X.A	.	.
Zm00001d012090	8	168029331	168034798	MVA, NMV	X.A	.	.
**WIDP GxE and Prediction GWA**							
Zm00001d028211	1	26522710	26532325	AA, X.A	MA, MD, MVA	.	.
Zm00001d001833	2	1623622	1634808	MD	X.A	.	.
Zm00001d003660	2	52983973	52990152	NMV	X.A	.	.
Zm00001d003661	2	52985264	52992526	NMV	X.A	.	.
Zm00001d039542	3	7733246	7739763	MD, MVA, NMV	MVA	High Phosphatidyl Choline1	hpc1
Zm00001d044130	3	220026982	220032889	TSA.RXSA, TSA.TCA	MA, MD, MVA	.	.
Zm00001d049894	4	51234927	51283694	MD, MVA	MD	.	.
Zm00001d053932	4	243765590	243773531	NMV	AA, X.A	.	.
Zm00001d021059	7	141817446	141823675	TSA.RXSA, TSA.TCA	MA, MD, MVA	Stearoyl-acyl-carrier-protein desaturase8	sacd8

Zm00001d021060	7	141819400	141826721	TSA.RXSA, TSA.TCA	MA, MVA	.	.
Zm00001d008900	8	24521219	24526917	NMV	MA, MD	.	.

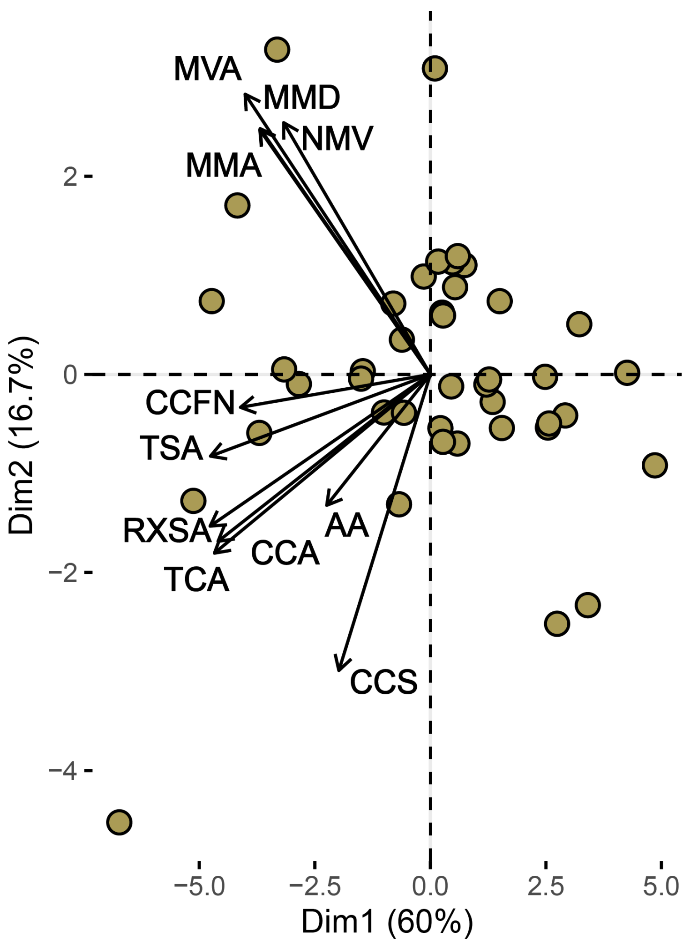

Supplemental Figure 1 PC1 vs PC2 loadings of main root anatomical traits for accessions from the Burton panel. Dimension 1 primarily delineates smaller root cross-sections from smaller root cross-sections. PC2 vs PC3 is shown in Figure 1A.

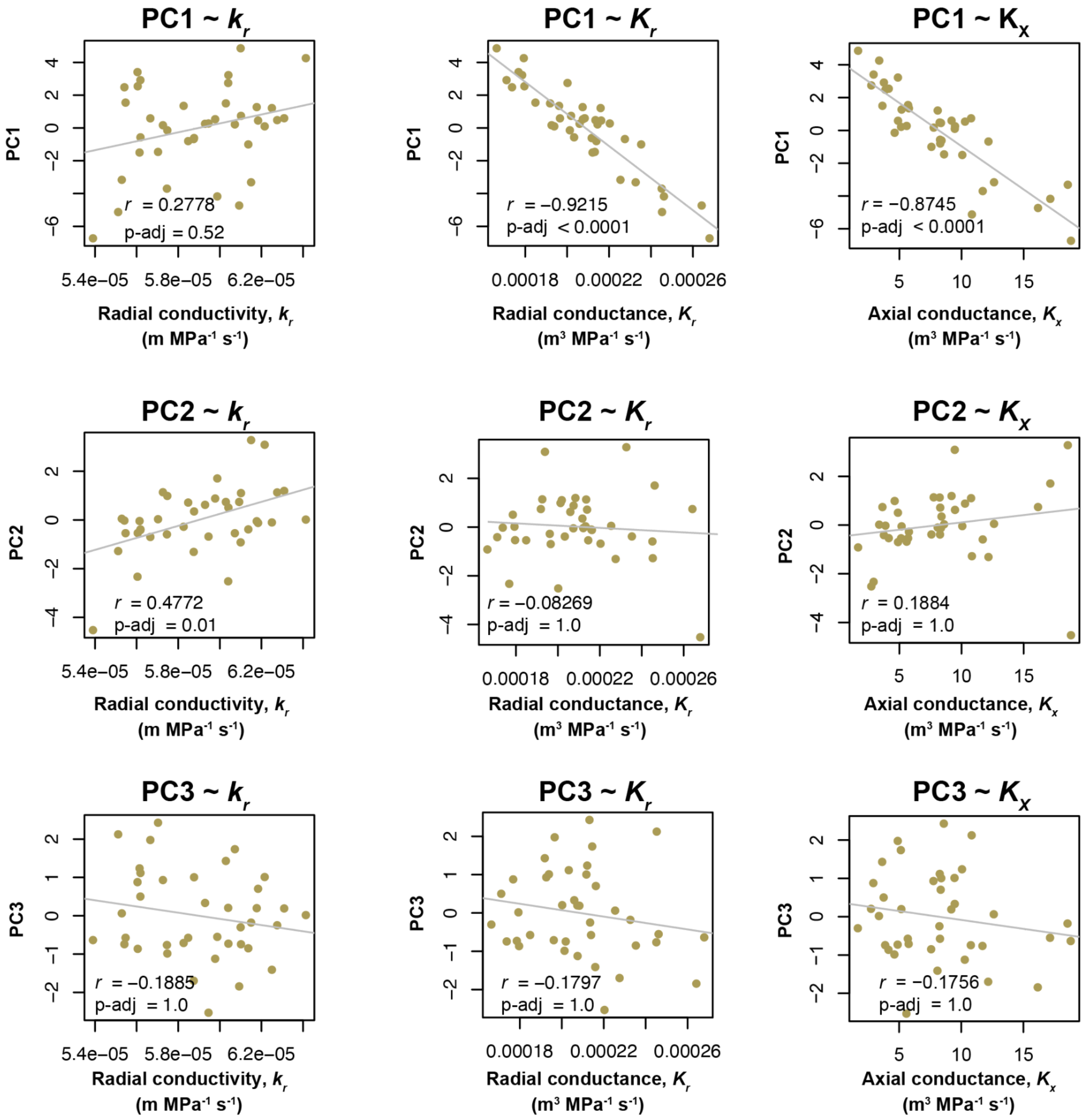

Supplemental Figure 2 Loadings of Burton panel anatomical PC1, PC2, and PC3 versus estimated radial conductivity (k_r) and radial and axial hydraulic conductance (K_r, K_x) of the same accessions. P-values are adjusted using the Holm-Bonferroni method.

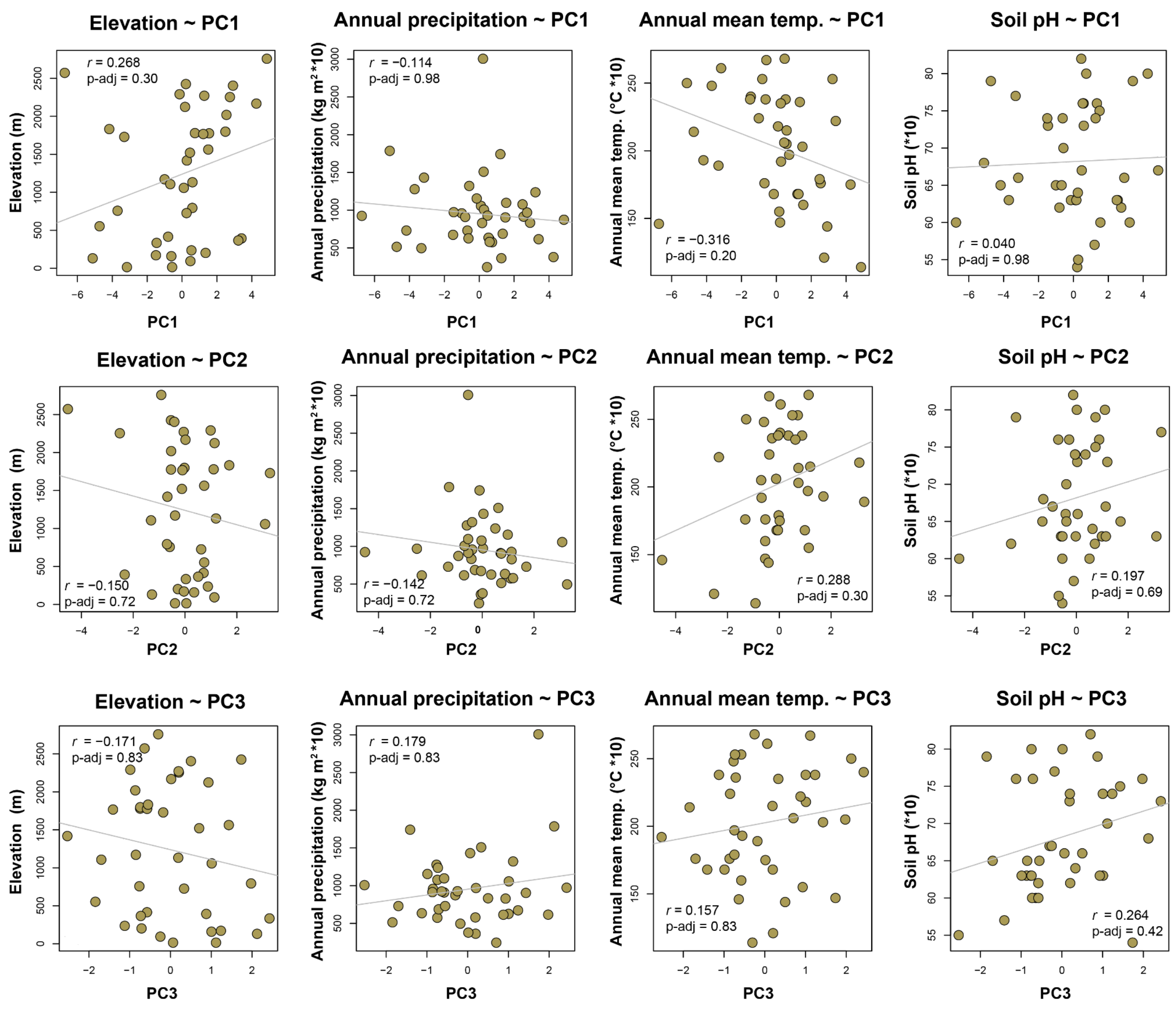

Supplemental Figure 3 Four core environmental aspects of Burton panel accessions' point of origin (elevation, annual precipitation, mean temperature and soil pH) versus loadings of Burton panel anatomical PC1, PC2, and PC3 of the same accessions. P-values for each PC are adjusted using the Holm-Bonferroni method.

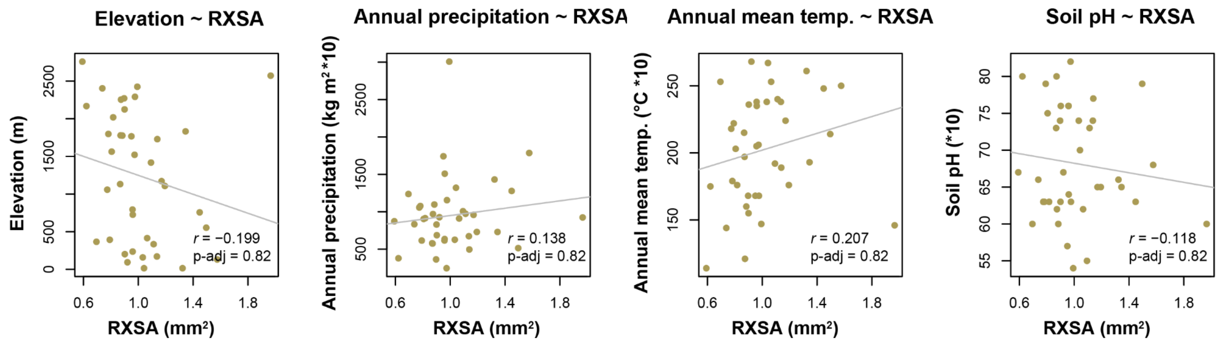

Supplemental Figure 4 Four core environmental aspects of Burton panel accessions' point of origin (elevation, annual precipitation, mean temperature and soil pH) versus root cross section area. P-values are adjusted using the Holm-Bonferroni method.

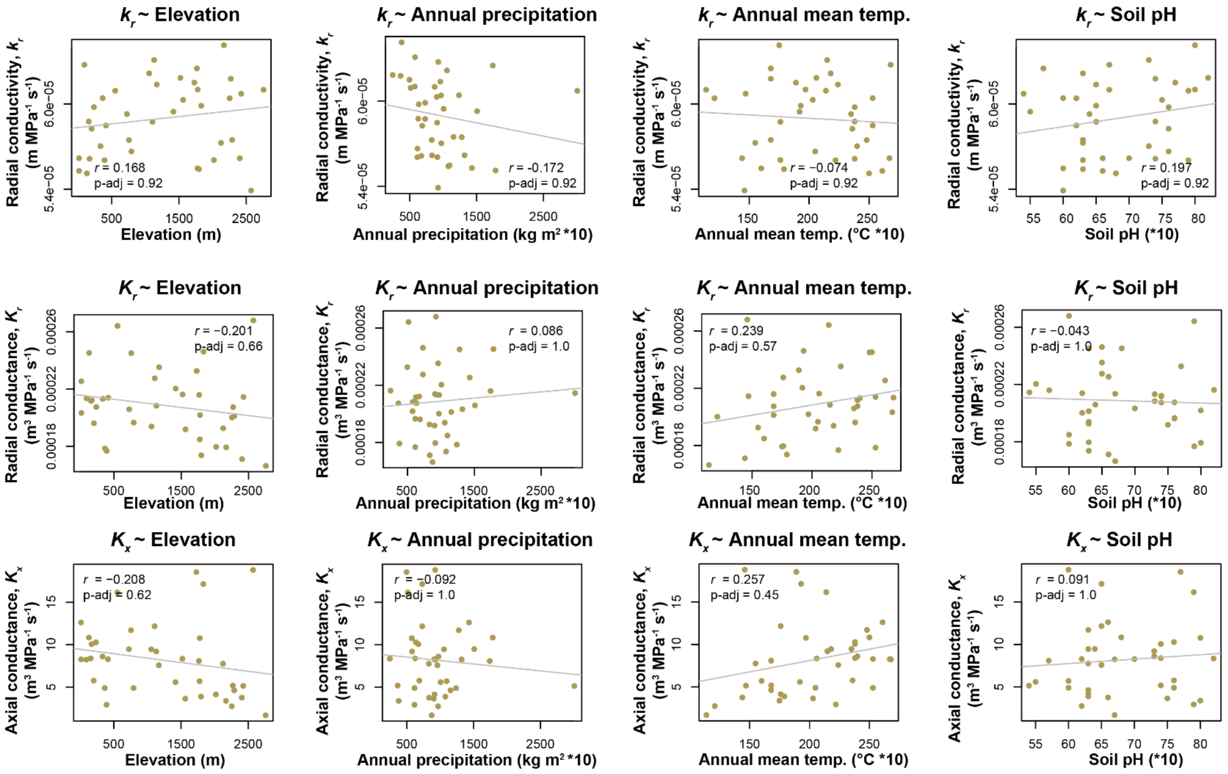

Supplemental Figure 5 Estimated radial conductivity (k_r) and radial and axial hydraulic conductance (K_r, K_x) of the Burton panel versus four core environmental aspects of accessions' point of origin (elevation, annual precipitation, mean temperature, and soil pH). P-values for each hydraulic scenario are adjusted using the Holm-Bonferroni method.

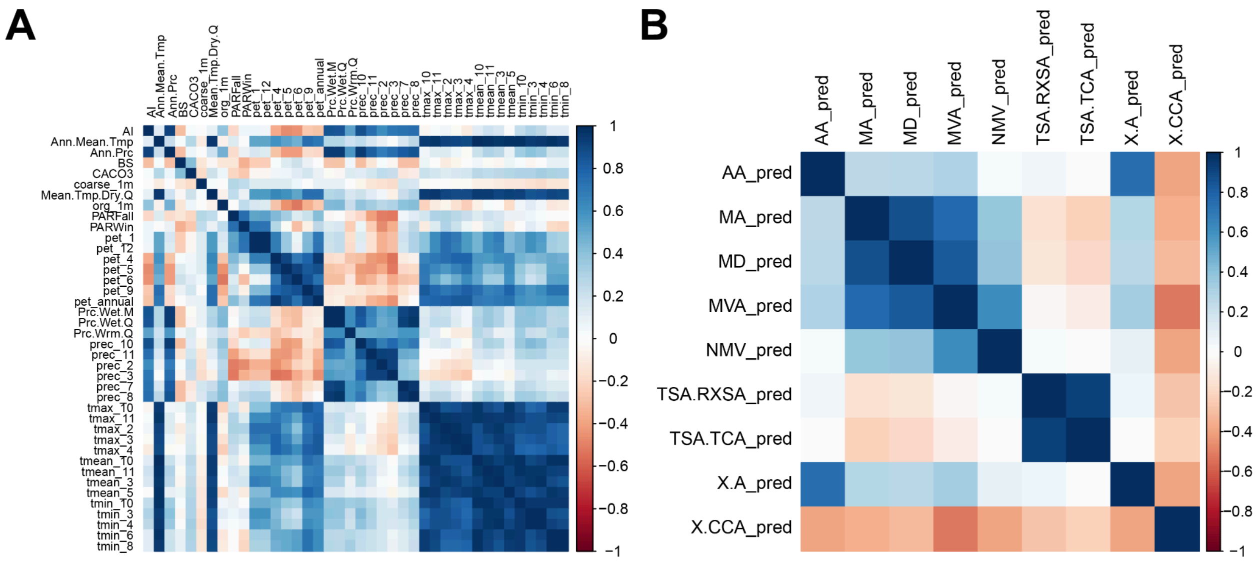

Supplemental Figure 6 Correlation plots of A) environmental descriptors used across all random forest models and B) random forest predicted anatomical traits of the CIMMyT panel. Blue colors indicate positive and red colors indicate negative correlations between variables.

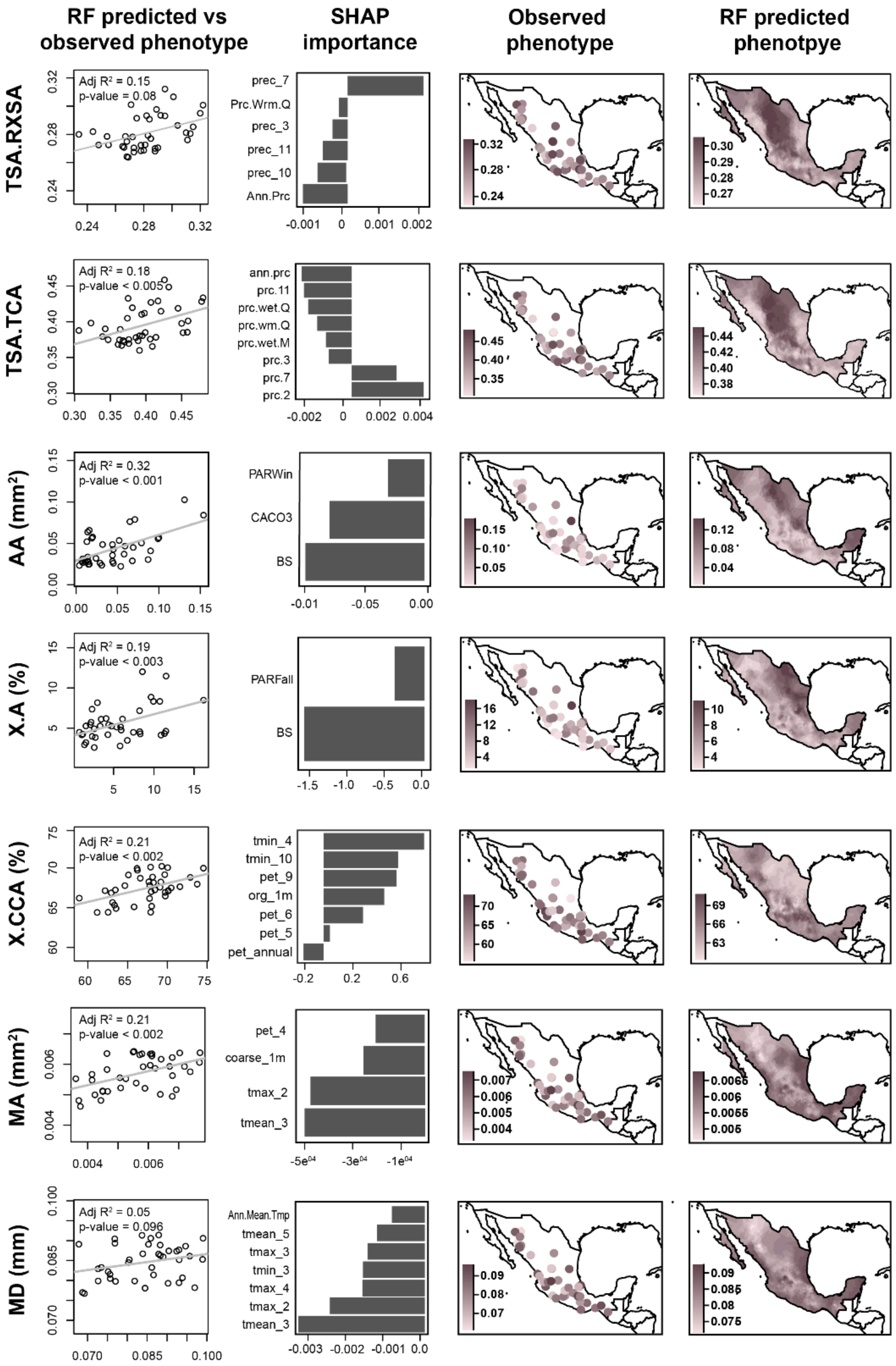

RF predicted vs observed phenotype
SHAP importance
Observed phenotype
RF predicted phenotpye
TSA.RXSA
Adj R² = 0.15
p-value = 0.08
prec_7
Prc.Wrm.Q
prec_3
prec_11
prec_10
Ann.Prc
TSA.TCA
Adj R² = 0.18
p-value < 0.005
ann.prc
prc.11
prc.wet.Q
prc.wm.Q
prc.wet.M
prc.3
prc.7
prc.2
AA (mm²)
Adj R² = 0.32
p-value < 0.001
PARWin
CACO3
BS
X.A (%)
Adj R² = 0.19
p-value < 0.003
PARFall
BS
X.CCA (%)
Adj R² = 0.21
p-value < 0.002
tmin_4
tmin_10
pet_9
org_1m
pet_6
pet_5
pet_annual
MA (mm²)
Adj R² = 0.21
p-value < 0.002
pet_4
coarse_1m
tmax_2
tmean_3
MD (mm)
Adj R² = 0.05
p-value = 0.096
Ann.Mean.Tmp
tmean_5
tmax_3
tmin_3
tmax_4
tmax_2
tmean_3

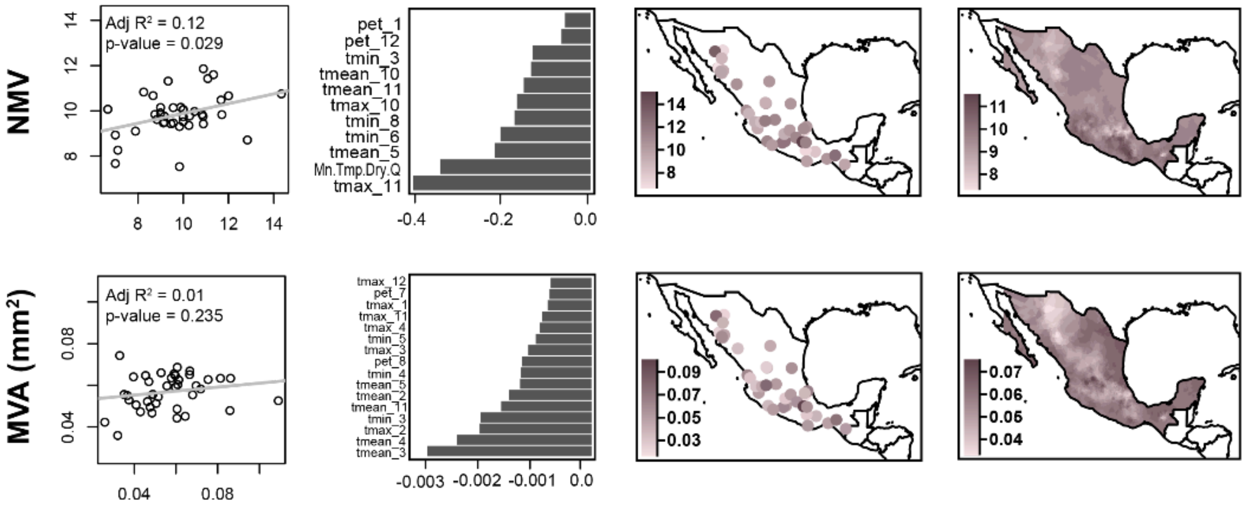

Supplemental Figure 7 A) Predicted random forest trait values vs observed trait values reported in Burton et al., 2013 used in model training and validation. Line is the coefficient of determination for all plotted points. B) SHAP values (SHapley Additive exPlanations) of environmental descriptors used in model training, describing the contribution of each environmental descriptor for each trained random forest model. C) Burton panel accession point of origin colored by reported trait observations used to train random forest models. D) Smoothed trait predictions as a function of the environment from constructed random forest models.

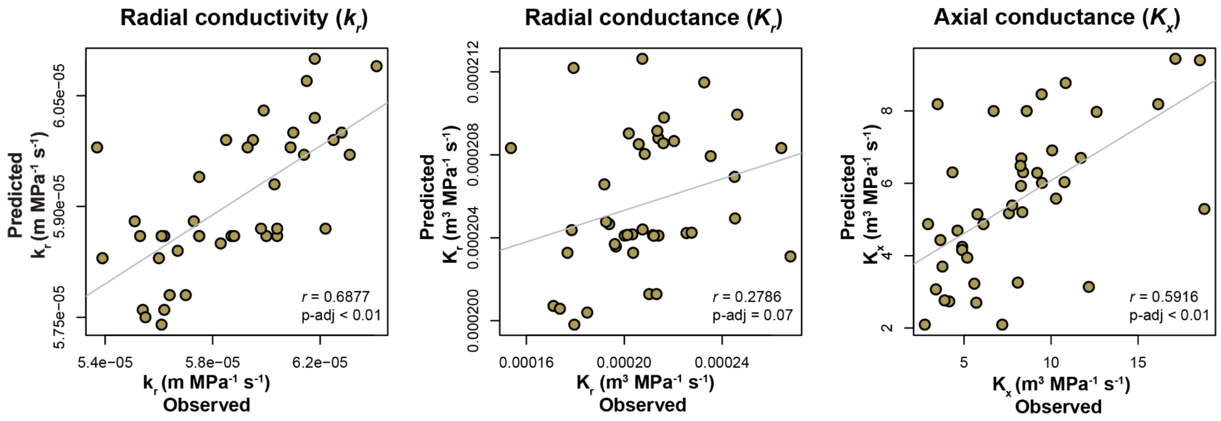

Supplemental Figure 8 Predicted vs observed radial conductivity (k_r), radial conductance (K_r), and axial conductance (K_x) for the Burton panel Mexican accessions. Radial conductivity is the same as shown in Figure 2D. P-values are adjusted using the Holm-Bonferroni method.

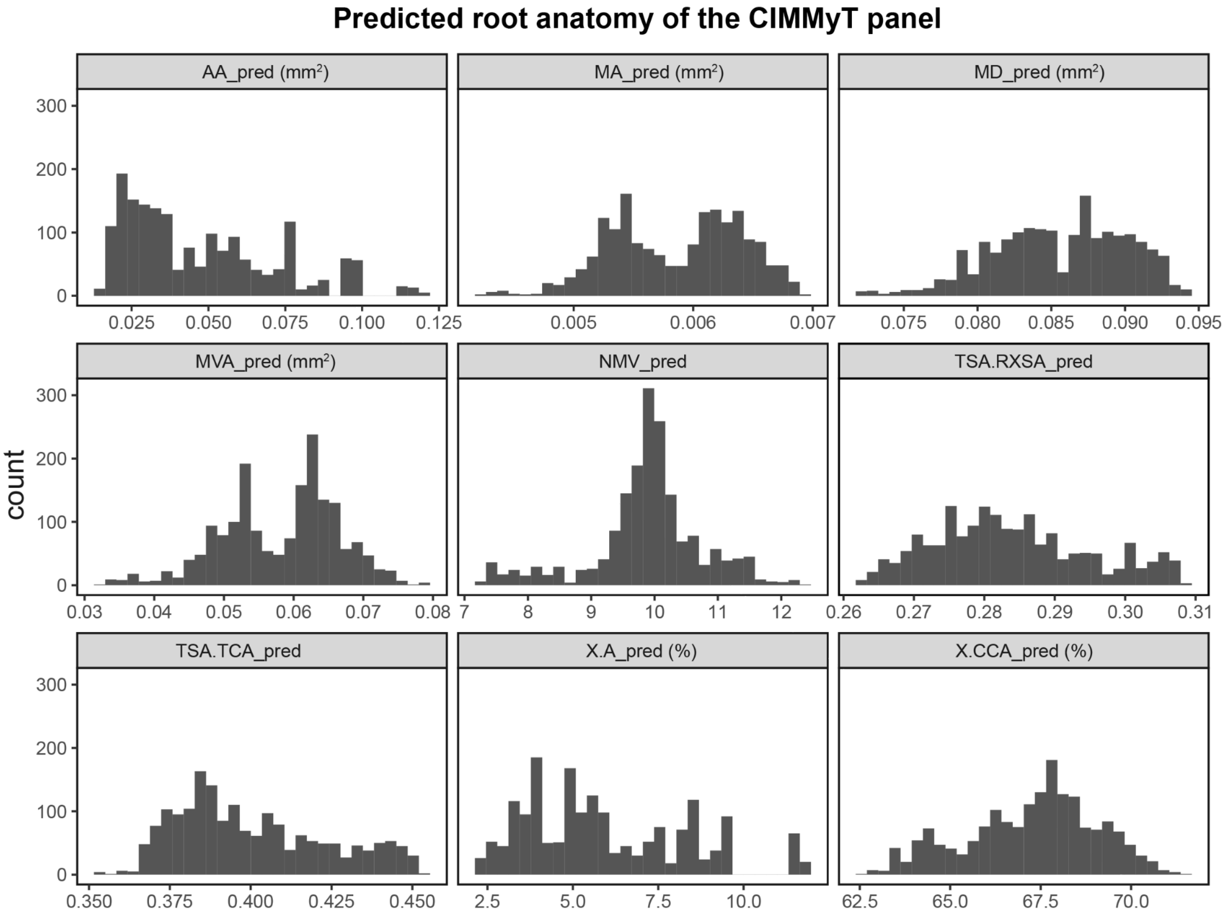

Supplemental Figure 9 Histograms of random forest anatomical trait predictions of the CIMMyT panel.

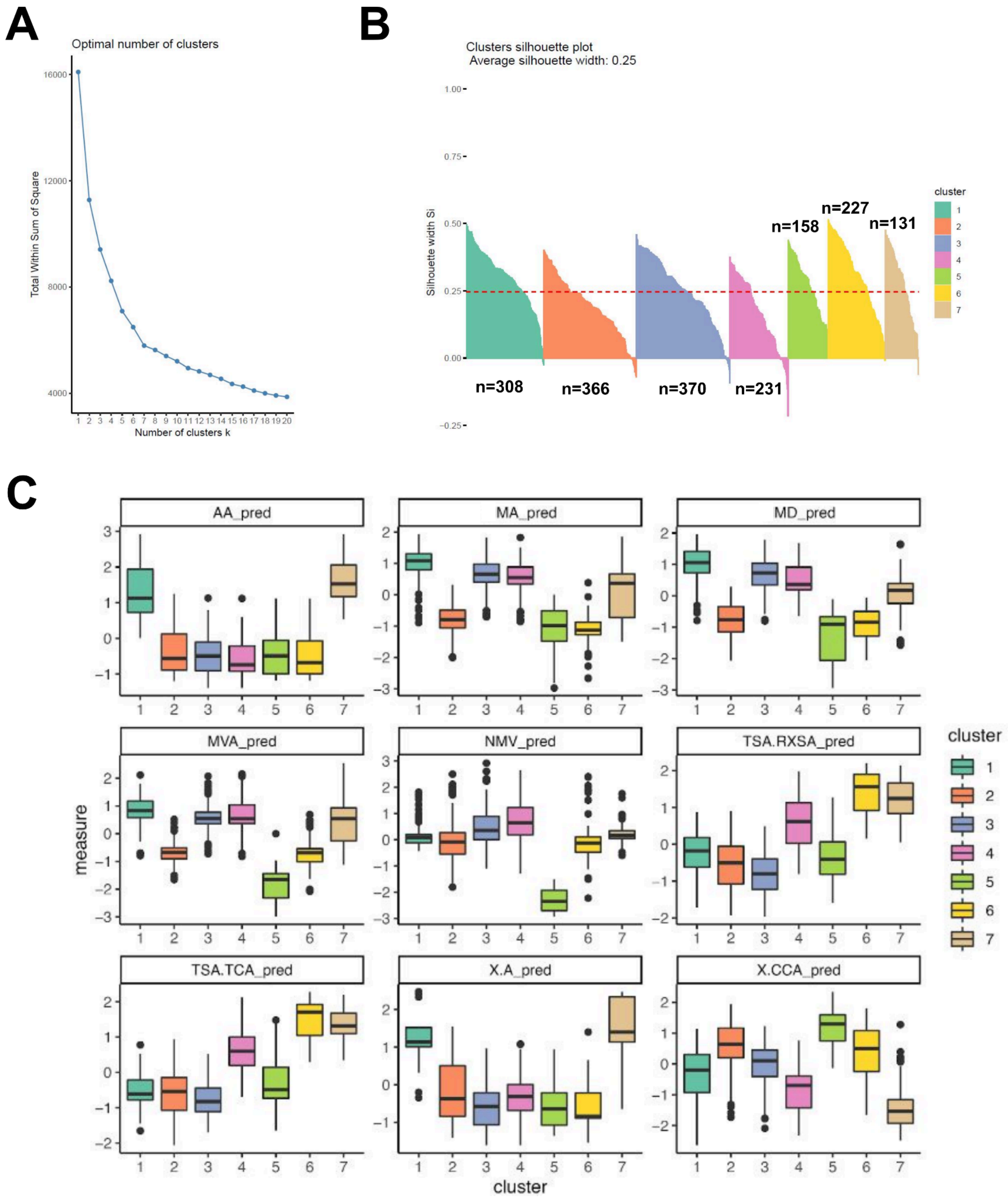

Supplemental Figure 10 A) Within cluster sums of squares for predicted traits of the CIMMyT panel for determining the optimal number of clusters used in partitioning against means (PAM) clustering. B) Cluster silhouettes summarizing the degree of separation between the clusters. The number of accessions assigned to each cluster is shown. C) Boxplots by cluster for all random forest predicted CIMMyT traits.

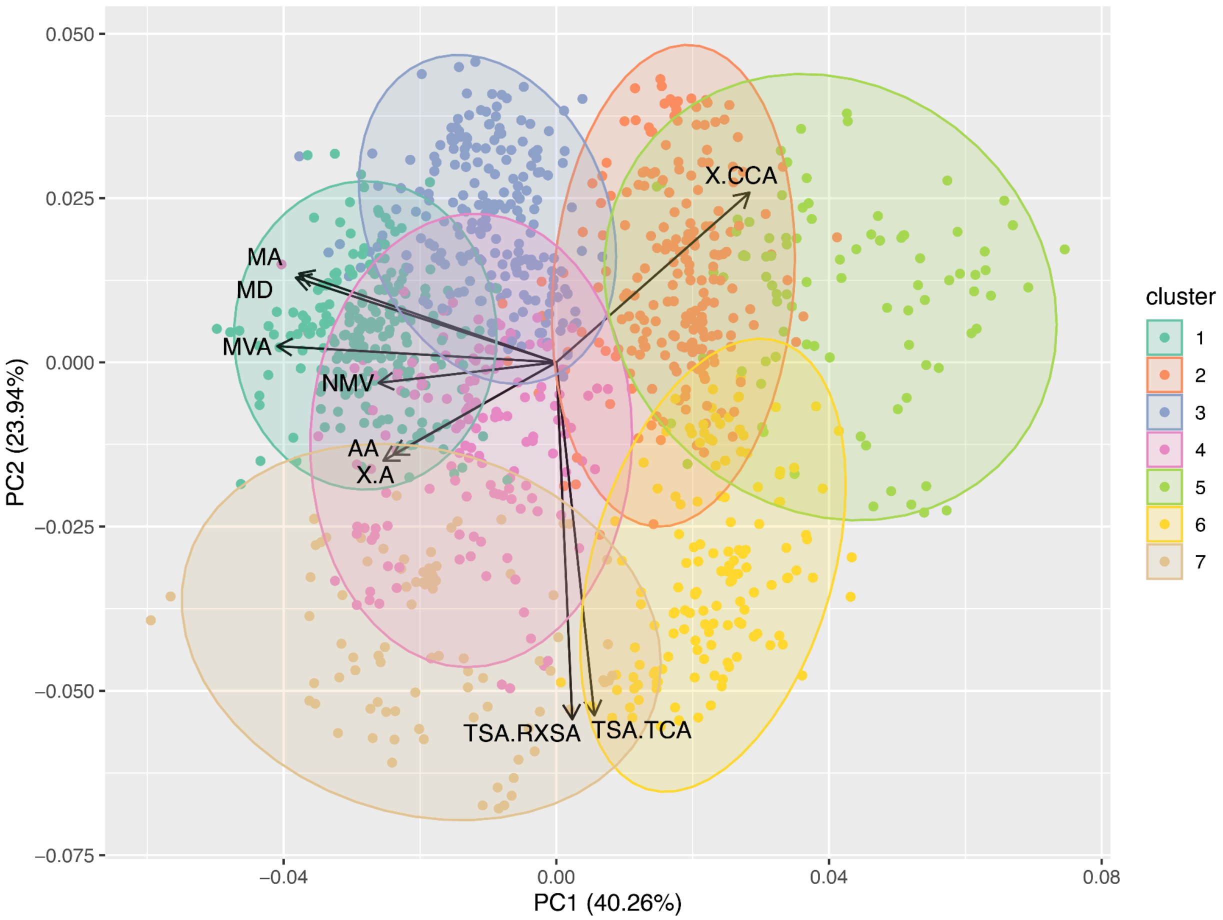

Supplemental Figure 11 PC1 vs PC2 of the CIMMyT panel random forest predicted traits used in PAM clustering. Clusters are defined by differences in cortical and metaxylem traits.

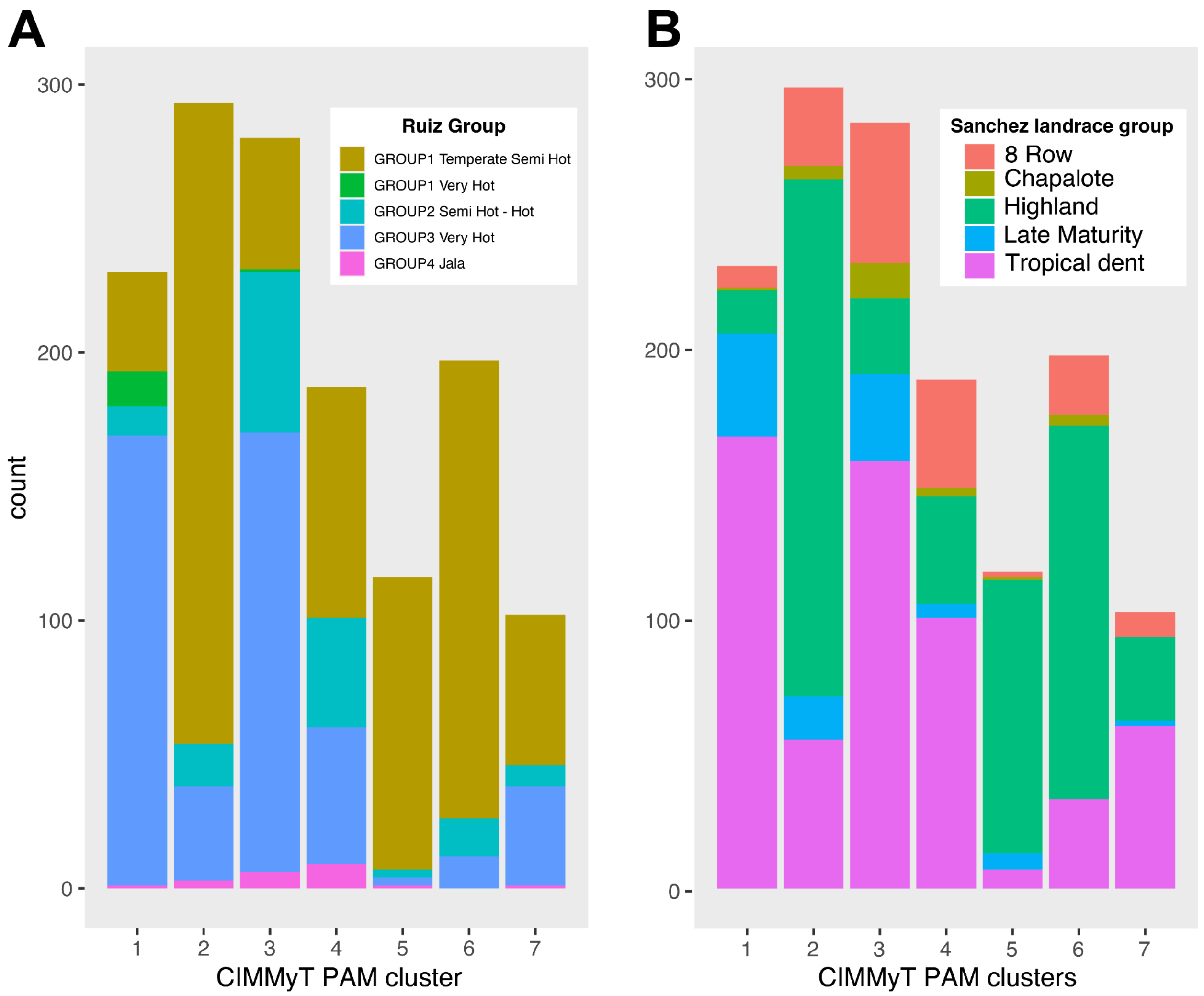

Supplemental Figure 12 Count of landrace designations present in the CIMMyT PAM clusters, grouped by previous A) environmental (Ruiz Corral et al. 2008) classifications and B) morphological-isozymatic (Sanchez G. et al. 2000). For each cluster, the primary morphological-isozymatic and environmental classification is reported in Supplemental Table 3.

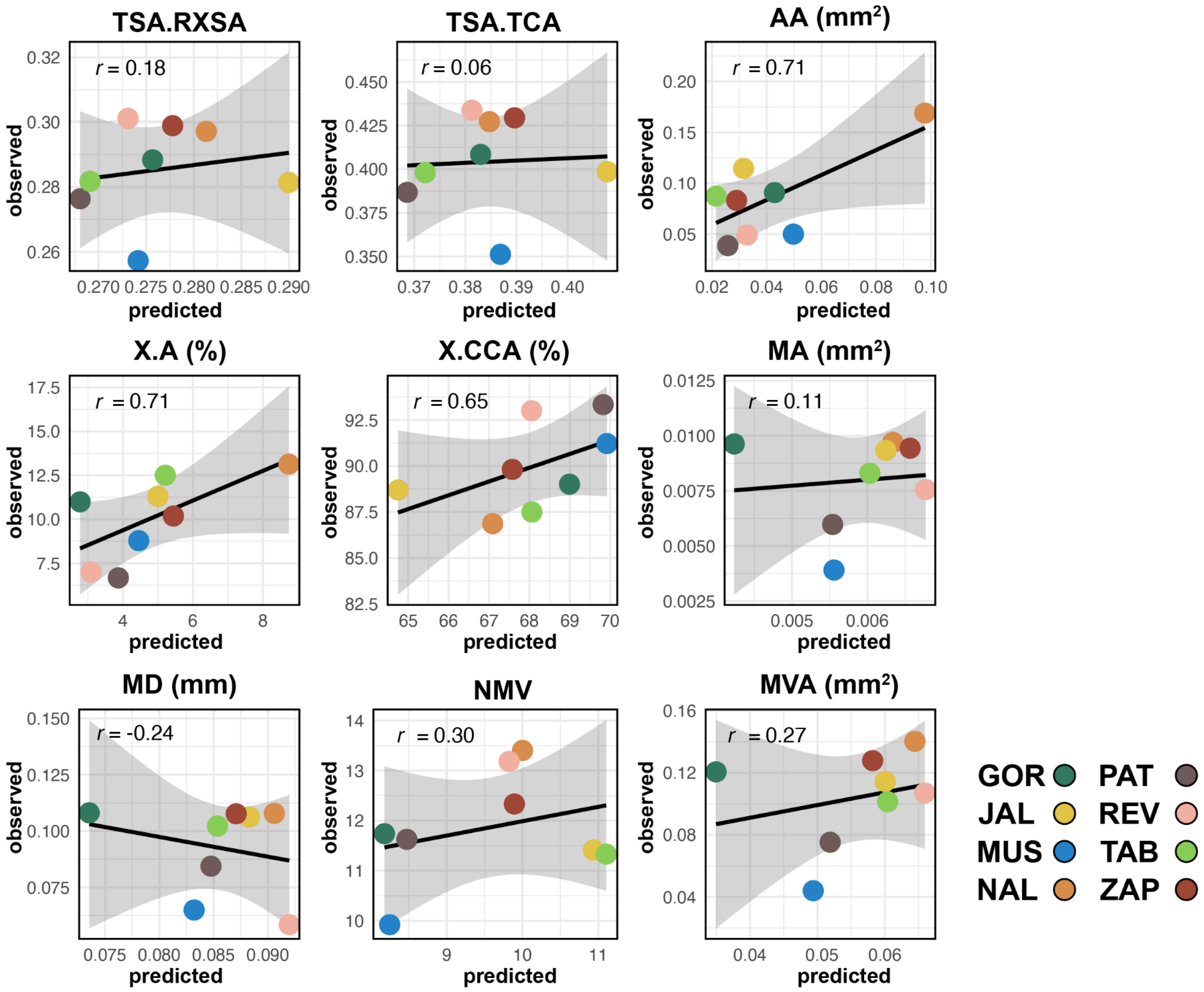

Supplemental Figure 13 Validation lines observed BLUPs of nodal root two and three from greenhouse grown plants vs RF trait predictions for all root anatomical traits. Root trait abbreviations are as described in Table 1.

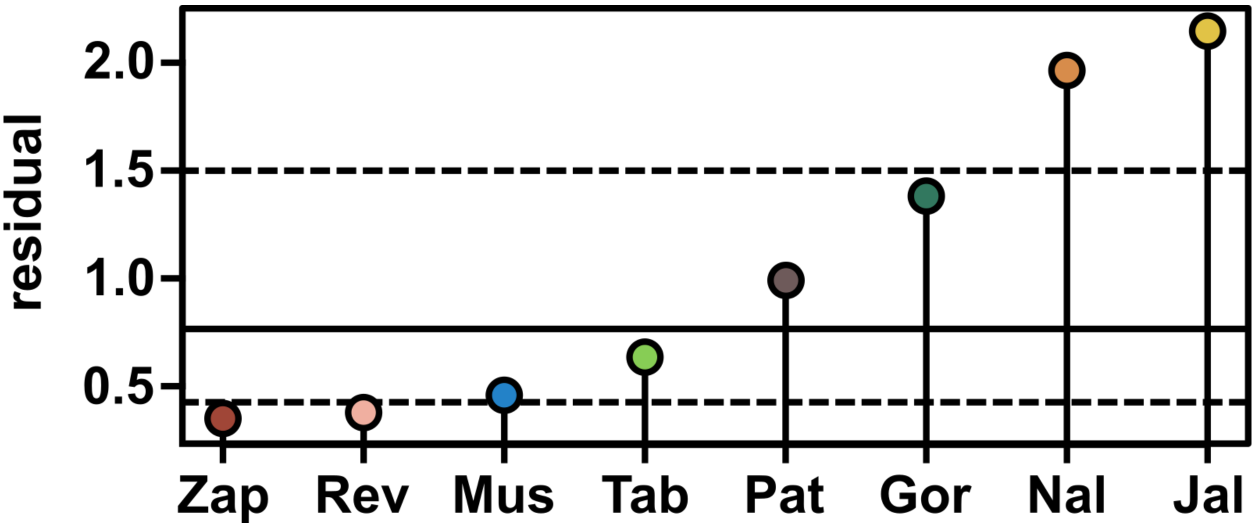

Supplemental Figure 14 Procrustes errors of comparing principal component analysis (PCA) of observed phenotypes of novel material grown in this study and random forest trait predictions (ordered according to the residual scores). Dashed and solid lines are the first, second, and third quartiles.

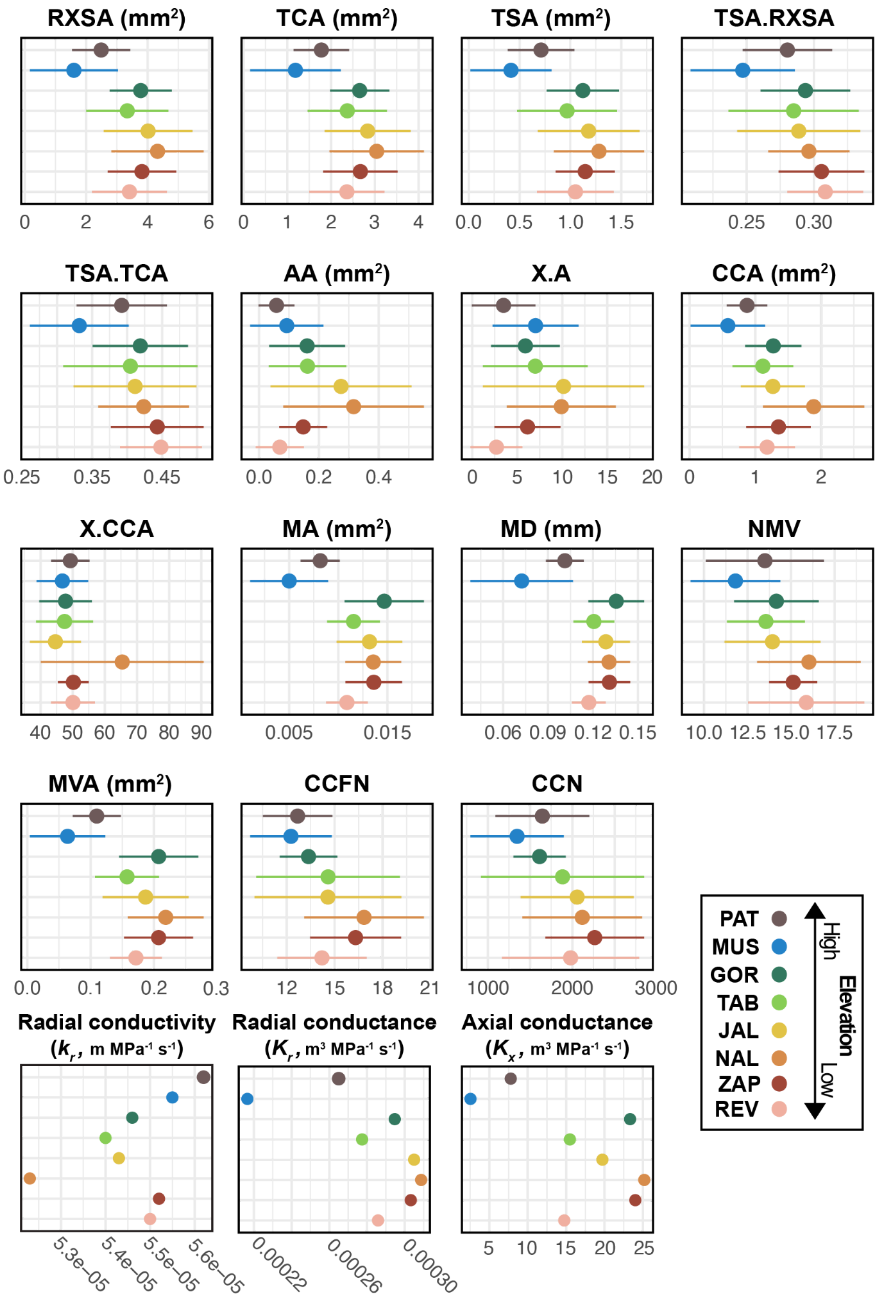

Supplemental Figure 15 Observed whorl three anatomical traits of novel material grown in this study, genotypes ordered by elevation. Each line represents the maximum and minimum trait value observed for a genotype with the circle as the genotype mean. Conductance and conductivity values are represented as the mean value for the genotype modeled by MECHA. Root trait abbreviations are as described in Table 1.

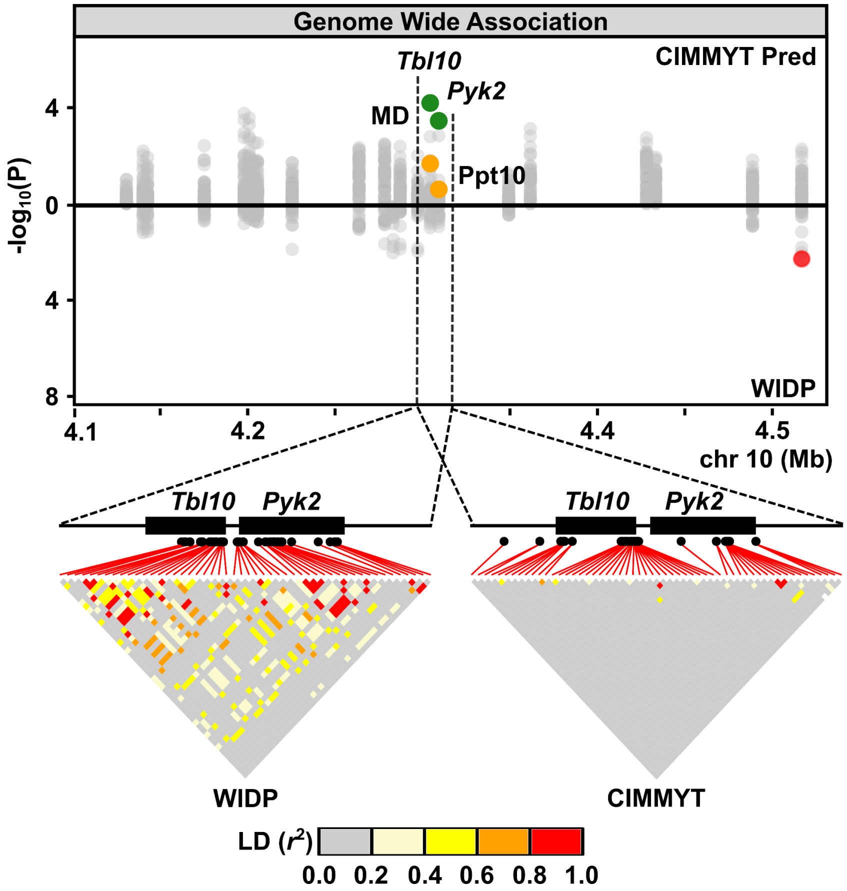

Supplemental Figure 16: Miami plot showing GWA support (-log$_{10}$P) for association with root anatomy for genes on a region of chromosome 10. Points above the x-axis show support for predicted phenotypes in the CIMMyT panel; points below the x-axis show support for observed phenotypes for the WIDP panel. Two genes (*Tbl10* and *Pyk2*) in the highlighted region show strong support for *mean metaxylem vessel diameter* (MD) in the CIMMyT panel, but no signal for the WIDP panel. Image below the Miami plot shows *Tbl10* and *Pyk2* genes in genomic context (CDS as filled boxes), SNP position (filled circles) and pairwise linkage disequilibrium (LD) for the CIMMyT and WIDP markers.
